# Molecular assembly of rhodopsin with G protein-coupled receptor kinases

**DOI:** 10.1038/cr.2017.72

**Published:** 2017-05-19

**Authors:** Yuanzheng He, Xiang Gao, Devrishi Goswami, Li Hou, Kuntal Pal, Yanting Yin, Gongpu Zhao, Oliver P Ernst, Patrick Griffin, Karsten Melcher, H Eric Xu

**Affiliations:** 1 Laboratory of Structural Sciences, Center for Structural Biology and Drug Discovery, Van Andel Research Institute, Grand Rapids, MI 49503, USA; 2 Department of Molecular Therapeutics, The Scripps Research Institute, Scripps Florida, Jupiter, FL 33458, USA; 3 VARI-SIMM Center, Center for Structure and Function of Drug Targets, CAS-Key Laboratory of Receptor Research, Shanghai Institute of Materia Medica, Chinese Academy of Sciences, Shanghai 201203, China; 4 The Cryo-EM Core, Van Andel Research Institute, Grand Rapids, MI 49503, USA; 5 Department of Biochemistry, University of Toronto, Toronto, Ontario M5S 1A8, Canada; 6 Department of Molecular Genetics, University of Toronto, Toronto, Ontario M5S 1A8, Canada; 7 Current address: Amgen, Thousand Oaks, CA 91320, USA

**Keywords:** rhodopsin, GPCR, GRK1, GRK5, Q41L

## Abstract

G protein-coupled receptor kinases (GRKs) play pivotal roles in desensitizing GPCR signaling but little is known about how GRKs recognize and phosphorylate GPCRs due to the technical difficulties in detecting the highly dynamic GPCR/GRK interaction. By combining a genetic approach with multiple biochemical assays, we identified the key determinants for the assembly of the prototypical GPCR rhodopsin with its kinase GRK1. Our work reveals that the regulatory G-protein signaling homology (RH) domain of GRKs is the primary binding site to GPCRs and an active conformation of the GRK1 kinase domain is required for efficient interaction with rhodopsin. In addition, we provide a mechanistic solution for the longstanding puzzle about the gain-of-function Q41L mutation in GRK5. This mutation is in the RH domain and increases the capacity of the GRK mutant to interact with and to desensitize GPCRs. Finally we present the principal architecture of a rhodopsin/GRK complex through negative stain electron microscopy reconstruction. Together, these data define the key components for the rhodopsin/GRK1 interaction and provide a framework for understanding GRK-mediated desensitization of GPCRs.

## Introduction

G protein-coupled receptors (GPCRs) comprise the largest receptor family in humans ^1^. Its members play fundamental roles in a broad spectrum of physiological processes and are involved in a plethora of diseases, including metabolic syndrome, neurodegenerative diseases, immune disorders and cancers^2, 3^. Extracellular signals activate GPCRs through ligand binding to induce a conformational change that favors the binding of heterotrimeric G proteins to initiate GPCR signaling^4, 5^. In many cases, the activated receptors need to be deactivated in a timely manner to maintain cellular homeostasis. G protein-coupled receptor kinases (GRKs) deactivate GPCR signaling by phosphorylating the intracellular loops and/or the C-tail of the receptor. The receptor is then recognized by arrestins, which block G-protein access and induce the internalization of the receptor-arrestin complex to shut down the signal transduction^6,7^. Since GRKs play pivotal roles in the desensitization of GPCR signaling, mutations of GRKs are associated with a variety of diseases, such as heart failure, Parkinson's disease and diabetes^8,9^. While most GRK mutations have detrimental effects, a gain-of-function mutation that is commonly found in African Americans, GRK5 Q41L, is of particular interest as it has a well-established protective effect against heart failure by enhancing β-adrenergic receptor desensitization^10^. However, how this mutation affects GRK5 activity remains unknown.

GRKs belong to the AGC family of serine/threonine kinases^7^. The GRK family can be further divided into three subfamilies based on sequence homology: the rhodopsin kinase subfamily (GRK1 and GRK7), the β-adrenergic receptor kinase subfamily (GRK2 and GRK3) and the GRK4 subfamily (GRK4, GRK5 and GRK6). All GRK family members share a common structural architecture, in which the kinase domain is inserted into a loop between the first 9 and the 10 α-helix of a regulatory G-protein signaling homology (RH) domain, followed by a variable carboxyl terminus. The RH core domain of GRKs contains ∼130 amino acids that form a bundle of nine helices (α1-α9), which is conserved in all regulatory G-protein signaling (RGS) proteins^11^. The kinase domain of GRKs contains a large C-terminal lobe and a small N-terminal lobe, closely related to those of other members of the AGC protein kinase family. Although the C-terminal domains of GRKs are least conserved, a common feature of the C-terminal domain is membrane binding^12^.

Similar to heterotrimeric G proteins and arrestins, GRKs recognize active GPCRs. The molecular mechanism of how these key regulators recognize the active receptors is a major focus of the GPCR field. Crystal structures of the β2-adrenergic receptor (β2AR)-bound Gs^13^, active rhodopsin (Rho) bound to a C-terminal Gα peptide^14^ and visual Rho-bound arrestin^15^ revealed that G protein and arrestin use similar but distinct strategies to interact with activated receptors. For instance, both Gs and arrestin recognize the outward movement of the cytoplasmic ends of the GPCR transmembrane (TM) helices. However, while G proteins mainly utilize the ligand-induced extension of TM5 and TM6 of β2AR for binding, visual arrestin (arrestin1) uses multiple discrete small patches, including TM7 and cytoplasmic helix 8 to interact with the activated Rho. Compared to GPCR/G-protein and GPCR/arrestin interactions, GPCR/GRK interactions are much weaker and much more dynamic, properties which have made the direct crystallization of a GPCR/GRK complex currently unattainable. Most knowledge on the receptor/GRK interaction is based on indirect assays, such as GPCR phosphorylation assays, and on molecular modeling based on limited structural information for GRKs and receptors.

Rho is a prototypical GPCR. It is responsible for visual signal transduction in rod cells, and has been extensively studied as a model for understanding GPCR signaling^16,17^. GRK1 phosphorylates light-activated Rho and initiates a series of events that lead to rapid quenching of the receptor signaling^18^. We had previously used the combination of a genetic approach with multiple biochemical assays to study the Rho/arrestin interaction^15^. To gain insight into GRK1-mediated Rho desensitization, we have used a similar strategy to dissect the Rho/GRK1 interaction. We discovered that the interaction of GRK1 with Rho is mainly mediated by the RH domain of GRK1 and requires an active conformation of the kinase domain. We also uncovered the mechanism by which the Q41L mutation of GRK5 enhances the desensitization of GPCR signaling and present an overall architecture of the Rho/GRK complex assembly through single-particle reconstruction from negative stain electron microscopy (EM) images.

## Results

### Establishment of an effective method to detect the transient interaction between rhodopsin and GRK1

**Figure 1:**
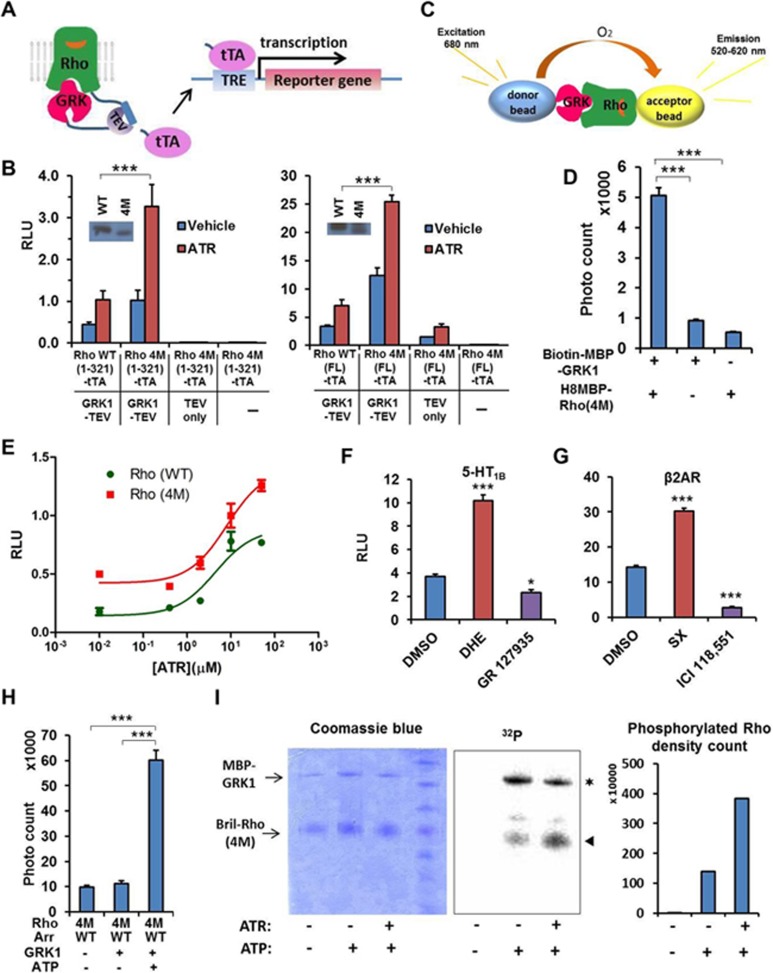
Establishment of methods for detecting the rhodopsin/GRK1 interaction. **(A)** Schematic diagram of a modified Tango assay for detecting the rhodopsin/GRK interaction. **(B)** Examination of the rhodopsin/GRK1 interaction in Tango assay. The protein expression levels of rhodopsin-TEV cleavage site-TetR fusion proteins were detected by anti-TetR antibody. RLU, relative luciferase units. ATR, 10 μM. **(C)** Schematic diagram of the AlphaScreen assay for detecting the rhodopsin/GRK1 interaction. **(D)** Examination of the rhodopsin/GRK1 interaction by AlphaScreen assay. **(E)** Dose response of ATR for both WT and 4M of rhodopsin in the Tango system. **(F)** Examination of GRK2/5-HT_1B_ interaction by the Tango assay. DHE 0.1 μM, GR 127935 1 μM. *P*-values are relative to the DMSO control. **(G)** Examination of GRK2/β2AR by the Tango assay. SX 1 μM, ICI 118 551 HCl 1 μM. Statistics to the DMSO control. **(H)** Examination of the rhodopsin-phosphorylation activity of GRK1 via indirect kinase assay. The activity is indirectly determined by measuring the phosphorylation-dependent interaction between rhodopsin and arrestin by AlphaScreen assay. **(I)** Direct kinase assay for the activity of MBP-GRK1. ^*^ indicates autophosphorylated MBP-GRK1, ▴ indicates phosphorylated rhodopsin. ^*^P < 0.05; ^**^P < 0.01; ^***^P < 0.001. ATR, all-*trans*-retinal; DHE, dihydroergotamine; NS, not significant; RLU, relative luciferase units; SX, salmeterol xinafoate.

The transient and dynamic nature of the GPCR/GRK interaction makes it difficult to use conventional methods such as co-IP and pull-down assays to gain insight into its molecular and structural basis. *In vivo* fluorescence or luminescence-based proximity assays (e.g., FRET or BRET) have been developed for detecting GPCR/GRK interactions^19^, yet their sensitivity is usually not sufficient to detect the weak association between the two components due to low signal-to-noise ratios^20^. The lack of an efficient method to detect this weak and transient interaction has become a hurdle for studying the GPCR/GRK interaction. A genetic approach called the Tango assay has been developed to detect GPCR/arrestin interactions *in vivo*^21^. This method converts a transient interaction into a stable and amplifiable reporter signal to record the activation of a receptor without interference from endogenous signaling pathways. We have successfully used this method to detect the interaction between Rho and visual arrestin, and to validate the crystal structure we determined for the Rho/arrestin complex^15^. To overcome the difficulty in detecting the weak Rho/GRK1 interaction, we therefore adopted the Tango assay. Specifically, we fused human GRK1 to the N-terminus of tobacco etch virus (TEV) protease, and human Rho via an optimized TEV protease cleavage site to a modified transcriptional activator tTA from the Tet-ON 3G system that is highly sensitive and more robust than the conventional tTA (see Materials and Methods for details) (Figure 1A). An interaction between Rho and GRK1 brings the TEV protease in the direct vicinity of the cleavage site to cut and release tTA, which allows tTA to enter the nucleus to activate reporter gene transcription. We first tested whether this method can detect the active conformation of Rho. While Rho is a light-activated GPCR, mutational studies showed that the light-activated conformation of Rho can be mimicked by certain mutations, e.g., the E113Q/M257Y mutation^22^. In addition, the introduced cysteine residues of a Rho N2C/N282C mutant protein from a disulfide bond that increases Rho's stability without affecting its activity^23^. We have successfully used the combination of these four mutations (4M) to study the active conformation of Rho in Rho/arrestin binding assays. Therefore, it is possible to use Rho 4M as a probe to test the recognition by GRK1. Our Tango data show that GRK1 interacts with both the wild-type (WT) and 4M versions of Rho, with 4M Rho interacting with GRK1 markedly stronger than WT Rho (Figure 1B), suggesting that GRK1 prefers the active conformation of Rho. Addition of the Rho ligand all-*trans*-retinal (ATR) further increased the activity (Figure 1B, red bar), again supporting the conclusion that GRK1 recognizes the active conformation of the receptor. Western blot analysis of Rho protein expression showed that WT and 4M constructs expressed at similar levels. In the context of a C-terminal tail truncation (after residue 321) of 4M receptor, the “TEV only” control (no fusion; Figure 1B, left panel lane 3) shows no activity, indicating that the interaction is highly specific to the GRK1-TEV fusion protein. In the context of full-length (FL) 4M receptor, the TEV control still retains residual activity that is below the signal from the FL WT receptor (Figure 1B, right panel lane 3). In all cases where the TEV construct is omitted (Figure 1B, lane 4 of both right and left panels), reporter gene activity is abolished, suggesting that the luciferase reporter activity depends on TEV enzyme activity. We also noticed that the interaction signals in the context of FL receptor are much stronger than for the C-tail truncation of Rho (Figure 1B). We reasoned that this is likely due to the flexibility of the C-tail of Rho, which increases the accessibility of the TEV cleavage site to the TEV enzyme nonspecifically, as seen from the relative high intrinsic activity of the TEV only control (Figure 1B). We also noticed that, in the context of FL Rho, the TEV only construct had a response to ATR induction and reasoned that this was because ligand induced a change in the receptor from a closed conformation to a more open state, which in turn made the C-tail more flexible and accessible to random cleavage by TEV protease.

We then used an *in vitro* AlphaScreen assay to validate the interaction between Rho and GRK1 and to test the importance of the Rho C-tail by an assay that is not affected by the tail's flexibility. In this assay, purified N-terminally His8-tagged Rho was immobilized to Ni-coated acceptor nano beads and purified N-terminally biotin-tagged GRK1 was immobilized to streptavidin donor beads. An interaction between Rho and GRK1 brings donor beads into close proximity of acceptor beads to allow a singlet oxygen-mediated transfer of excitation energy from donor to acceptor to generate an amplified luminescence signal (Figure 1C). As shown in Figure 1D, the interaction of 4M Rho with GRK1 was readily detected in the AlphaScreen assay (Figure 1D). We noticed that the interaction signal for the Rho/GRK1 interaction is much lower than for the Rho/arrestin interaction (compare Figure 1D-1H). This suggests that GRK1 interacts weakly with active Rho *in vitro*, and explains why so many methods have failed to detect this transient interaction.

To estimate how our Tango system responds to the Rho ligand ATR, we performed a dose response study of ATR for both WT and 4M Rho. The data show that Rho starts to respond to ATR in the Tango assay at a concentration of 1 μM and gives maximal response at 50 μM (Figure 1E). ATR concentrations higher than 50 μM caused a toxic effect on cells, and thus in most of our studies we used ATR at a concentration of 10 μM, a mid point in the dose-response curve. We further validated the Tango assays for GPCR/GRK interactions by determining the ligand effects on GRK2 recruitment by two other GPCRs: the serotonin receptor 5-HT_1B_ and the β2AR. For 5-HT_1B_, its agonist dihydroergotamine provided a 2.5-fold induction and the antagonist GR 127935 slightly inhibited 5-HT_1B_/GRK2 interaction when compared to the basal activity of the vehicle control (Figure 1F). Similarly, for β2AR, its agonist salmeterol xinafoate also gave a two-fold induction and its antagonist ICI 118 551 strongly inhibited the basal β2AR/GRK2 interaction (Figure 1G). Together, these data demonstrated that the Tango system provides a sensitive assay for the GPCR/GRK interaction and can be used to monitor the ligand response of GRK recruitment by GPCRs.

We also developed an indirect method without radioactive ^32^P labeling to examine the kinase activity of purified GRK1 protein based on the interaction between arrestin and GRK-phosphorylated Rho. It has been established that WT arrestin requires phosphorylated active Rho for tight binding, even if the receptor is in the active form^24^. Consistently, WT arrestin shows very limited binding to activated Rho (4M) (Figure 1H, lane 1). Adding our purified GRK1 without ATP to the reaction did not significantly increase the interaction, yet addition of purified GRK1 together with ATP greatly increased the interaction between WT arrestin and activated Rho (Figure 1H, lane 3), suggesting that the purified GRK1 indeed phosphorylates the activated Rho (4M). Finally, we used a direct kinase assay with radioactive ^32^P-γ-ATP to validate observations from the indirect kinase assay. We found that without ATP, there was no phosphorylation of the Rho (4M) substrate, whereas addition of ATP resulted in phosphorylation of the Rho (4M) substrate and addition of the ligand ATR further increased the phosphorylation by three-fold (Figure 1I). We also noticed phosphorylation of GRK1 itself, suggesting that the purified GRK1 protein has autophosphorylation activity.

### The rhodopsin/GRK1 interaction is mainly mediated by the RH domain of GRK1

**Figure 2:**
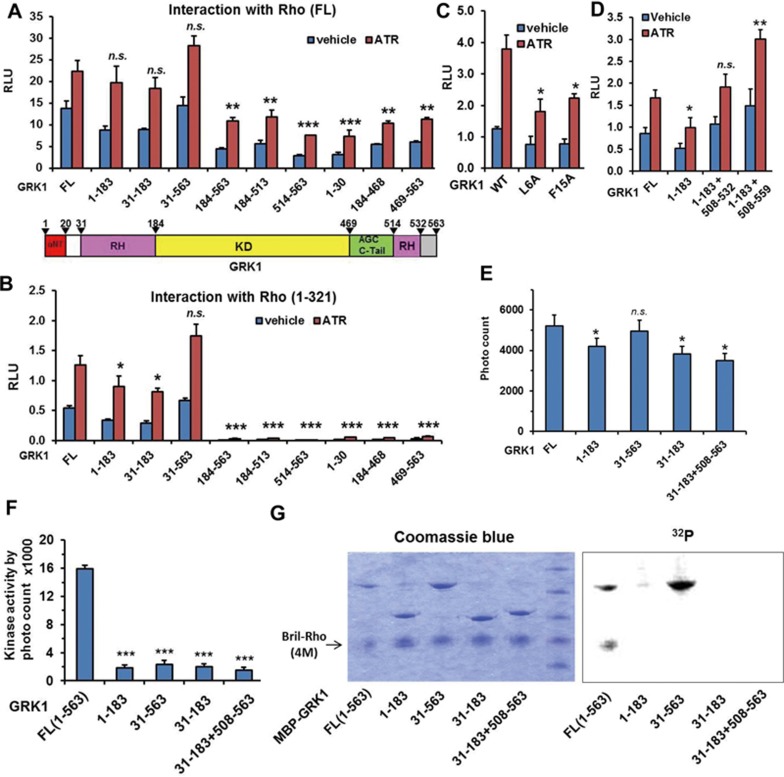
Mapping of the rhodopsin interaction domain of GRK1. **(A)** Domain analysis of the receptor interaction domain of GRK1 in the context of full-length rhodopsin by Tango assay. **(B)** Domain analysis of the receptor interaction domain of GRK1 in the context of C-tail truncated rhodopsin (residues 1-321) by Tango assay. **(C)** Analysis of mutations of the very N-terminus of GRK1 for rhodopsin (1-321) interaction by Tango assay. **(D)** Analysis of the intact RH domain for rhodopsin (1-321) interaction by Tango assay. Statistics were referred to the FL GRK1. **(E)** Validation of the rhodopsin/GRK1 interaction by AlphaScreen assay. Statistics were referred to the FL GRK1. **(F)** Indirect kinase activity assay of GRK1 truncations. The activity is indirectly determined by measuring the phosphorylation-dependent interaction between rhodopsin and arrestin by AlphaScreen assay. **(G)** Direct kinase assay of GRK1 truncations. ^*^P < 0.05; ^**^P < 0.01; ^***^P< 0.001. NS, not significant (differences relative to FL GRK1).

The ability of our Tango system to detect the weak interaction between the membrane protein Rho and its cytoplasmic partner GRK1 enabled us to map the receptor interaction domain of GRK1. GRK1, like all members of the GRK family, consists of a short N-terminal helix (αNT), which is only visible in certain GRK structures, followed by the RH core domain (RH helices 1-9), the conserved kinase domain with the AGC C-tail, the RH extension domain (helix 10) and the non-conserved C-terminus. We first analyzed the interaction between FL Rho and truncated GRK1. While fragments encompassing the RH core domain (GRK1 residues 31-183) retained most of the receptor interaction activity, all other truncations still elicited considerable levels of reporter signals (Figure 2A).

Because the interaction of GRKs with GPCRs requires the active conformation of the receptor, typified by an opening of the receptor TM core at the cytoplasmic side, we then focused on the highly specific interaction of GRK1 with the Rho core (residues 1-321), which includes cytoplasmic helix 8 but lacks the Rho C-tail (Figure 2B). In this context, the N-terminal GRK1 fragment (residues 1-183) retained about 70% of the receptor interaction signal relative to FL GRK1, while all C-terminal fragments lacking the RH core domain, including the kinase and C-terminal RH extension domains in combination with or without the GRK1 C-tail, showed no receptor interaction activity (Figure 2B). These data can be more clearly viewed when all domain activity was plotted as percentile activity of FL GRK1 (Supplementary information, Figure S1). A western blot of all GRK1 constructs showed that all GRK1 fragments were expressed; however, the expression levels varied from fragment to fragment (Supplementary information, Figure S2A, left panel). In particular, fragments mainly encompassing the kinase domain (184-468, 184-513 and 184-563) showed a much lower expression level than FL and other fragments. To rule out the possibility that the lack of interaction of the kinase domain fragments is due to the low expression levels, we titrated the expression levels of all constructs to a similar level by changing the amount of DNA in the transfection (Supplementary information, Figure S2A, right panel). To investigate the correlation of the Tango activity and the amount of transfected DNA, we carried out a dose-response curve of the amount of transfected DNA with the Tango activity for both GRK1 and Rho. The data showed that for both GRK1 and Rho, between 1 and 30 ng per well, the Tango signal linearly correlated with the amount of transfected DNA (Supplementary information, Figure S2B). In our Tango assay, we transfected DNA at ∼10 ng per well, which is in the middle part of the linear dose response. Thus, the titration allowed us to adjust DNA amounts to yield similar protein expression levels. Having established these transfection conditions, we used DNA ratios that resulted in approximately equal protein levels for all constructs (deletions/wild type) in the Tango assay to reevaluate the domain mapping results. The data show that, similar to the results obtained with equal amounts of DNA transfected (Figure 2B), the receptor interaction is mainly mediated by the RH domain (Supplementary information, Figure S2C). To avoid potential interference of protein expression levels on Tango assay activities, we also titrated the expression levels of the GRK1 point mutant construct to a similar level and transfected DNA at the corresponding ratios. A western blot of all key GRK mutant fragments and additional truncations used in this study is shown in Supplementary information, Figure S3. These results suggest that the observed lack of interaction was therefore not due to low expression levels of the deletion constructs and further support that the RH domain of GRK1 is the major binding interface with Rho.

The N-terminus of GRK1 contains the αNT helix (residues 1-20), which is conserved among all members of the GRK family, and has been proposed to play a crucial role in receptor binding based on its requirement for receptor phosphorylation^25^. Our extensive domain mapping data show that deletion of the αNT does not significantly affect receptor interaction (Figure 2B; Supplementary information, Figure S1). For instance, GRK1 (31-183) contains the RH core domain but lacks αNT, yet has a similar binding capacity as GRK1 (1-183), with both constructs retaining about 70% of the binding capacity of FL GRK1 (Figure 2A and 2B). Consistent with this result, GRK1 (31-563) differs from FL GRK1 only by the absence of the αNT-containing N-terminus, yet it retains full binding capacity relative to non-truncated GRK1 (Figure 2B; Supplementary information, Figure S1). In addition, the fragment comprising the N-terminal 30 amino acids of GRK1 (GRK1 (1-30)) did not show any interaction with the receptor (Figure 2B). Collectively, these data indicate that the very N-terminus is not needed for receptor binding. Further evidence comes from a mutation study of residues that were proposed to interact with αNT to form the so-called active conformation. The structure of GRK6 in which αNT was resolved shows that V477 of the AGC C-tail (V479 in GRK1) and R190 of the β3-strand of the kinase N-lobe of GRK6 (R194 in GRK1) form key interactions with αNT to hold αNT in a specific position believed to be the active conformation for receptor docking^26^. However, mutations of V479 and R194 in GRK1 did not inhibit Rho binding (Supplementary information, Figure S4A). Similarly, mutations of L4 and Y13 of GRK2 have been shown to abolish Rho phosphorylation based on a GPCR/GRK2 docking simulation study^20^. We therefore asked whether mutations of the corresponding residues in GRK1 (L6A and F15A, respectively) affect Rho binding. Our binding data revealed that these mutations did reduce receptor interaction but did not totally abrogate receptor binding (Figure 2C).

Since the N-terminal RH core domain contains most, but not all, of the receptor binding capacity (∼70%), we asked whether a complete RH domain (core domain plus C-terminal extension) has full receptor interaction capacity. As shown in Figure 2D, Supplementary information, Figure S1, constructs in which the RH core domain (helices α1-9) is fused with the C-terminal extension domain (helix α10 with or without the C-tail) by deletion of the kinase domain (1-183 + 508-559 and 1-183 + 508-532) have full receptor interaction capacity (≥ 100%, Figure 2D).

We then used the *in vitro* AlphaScreen assay to validate these observations. Similar to our observations using the Tango assay, the purified N-terminal domain of GRK1 (1-183) had about 70-80% receptor binding activity (Figure 2E). GRK1 (31-183) that has a further deletion of the very N-terminus including αNT had almost the same capacity as GRK1 (1-183). Consistently, GRK1 (31-563) had almost the same receptor binding activity as the FL protein. GRK1 (1-183 + 508-559), which had full activity in Tango assay, was difficult to express and purify. However, we could purify GRK1 (31-183 + 508-563) and show that it retained about 70% of the receptor binding capacity (Figure 2E). Together, these results indicate that the RH domain of GRK1 mediates the major interaction with Rho.

We next asked whether the RH domain-mediated receptor interaction is conserved within the GRK family. We chose GRK1, GRK2 and GRK5 to represent each subfamily, and tested their interaction with Rho, β2AR, 5-HT_1B_ and the class B GPCR parathyroid hormone receptor (PTH1R). GRK1 and GRK2 both showed a similarly strong interaction with Rho (Supplementary information, Figure S4B). For β2AR, PTH1R and 5-HT_1B_, GRK2 showed the strongest interaction signal, consistent with the observation that GRK2 is the predominant kinase for desensitizing a broad spectrum of GPCRs. Since GRK2 is the native partner for most GPCRs, we focused on GRK2 for the domain analysis. Consistent with the observation for Rho/GRK1, GRK2 (1-184) comprising the RH core domain contains almost full capability for interacting with β2AR, PTH1R and 5-HT_1B_ (Supplementary information, Figure S4C). Moreover, a further deletion of the first 27 amino acids (generating GRK2 (28-184)) does not affect the receptor interaction. One distinct feature of GRK2 is the pleckstrin homology (PH) domain at its C-terminus. The PH domain has the ability to bind to Gβγ and may help anchor GRK2 to the membrane for receptor binding. As shown in Supplementary information, Figure S4D, deletion of the C-terminal PH domain (GRK2 (1-547)) did not affect receptor binding ability. This result suggests that in addition to the PH domain, GRK2 may use additional domains for membrane binding. A sequence homology analysis suggests that a cluster of positively charged residues at the N-terminus of GRK2 may also participate in membrane binding^27^.

Our observation that the very N-terminus of GRK1 is dispensable for receptor binding was unexpected, as numerous studies suggested that this region is critical for receptor binding and phosphorylation^28,29^. Since most predictions for receptor binding were based on the fact that deletions or mutations of αNT inhibit receptor phosphorylation, we utilized the assay described in Figure 1H to examine whether N-terminally truncated GRK1 still has the ability to stimulate the phosphorylation-dependent interaction between receptor and arrestin. All GRK1 constructs that lack the kinase domain (1-183, 31-183 and 31-183 + 508-563) failed to stimulate the Rho-arrestin interaction, indicating that this assay indeed detects GRK1 kinase activity (Figure 2F). Very interestingly, GRK1 (31-563), which contains the intact kinase domain and binds activated Rho (Figure 2B and 2E) but lacks the very N-terminus, lost the ability to stimulate the phosphorylation-dependent interaction between Rho and arrestin (Figure 2F). This is consistent with reports that this region is crucial for receptor phosphorylation. We used the direct kinase assay to validate these observations. The data show that indeed deletion of the first 30 residues (generating GRK1 (31-563)) abolished the ability of GRK1 to phosphorylate Rho; however, surprisingly, we found this deletion has no effect on its autophosphorylation activity (Figure 2G). These data suggest that while the very N-terminus of GRK1 is not needed for receptor binding, it is essential for receptor phosphorylation.

### An active conformation of the kinase domain of GRK1 is needed for receptor interaction

**Figure 3:**
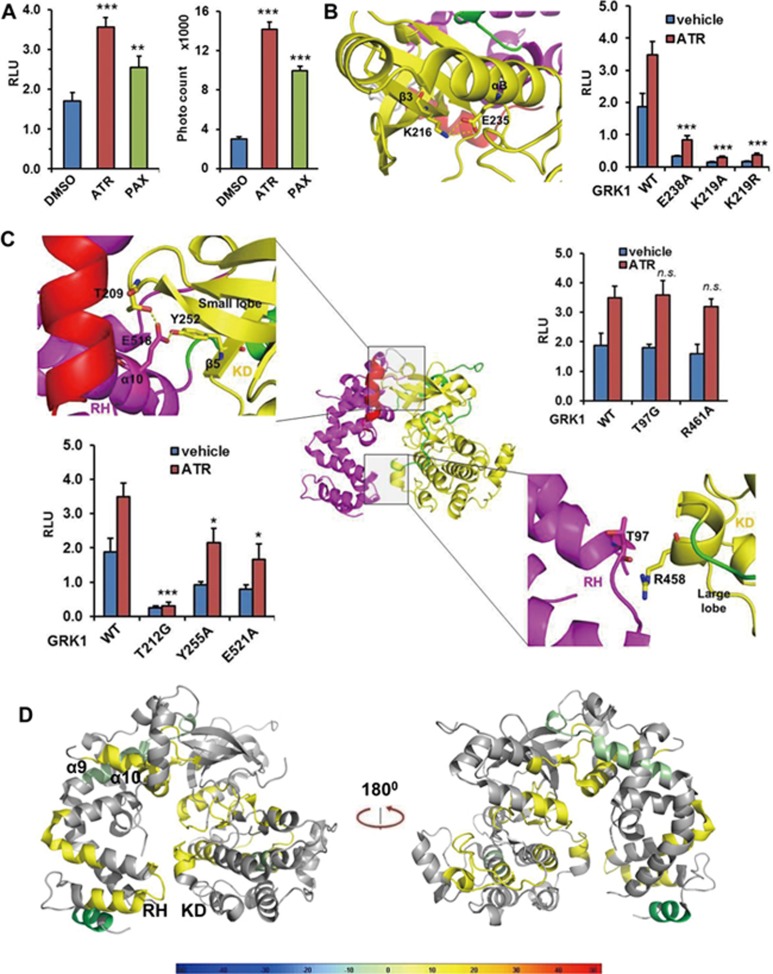
An active conformation of the kinase domain is needed for rhodopsin (1-321) interaction. **(A)** Paroxetine increases the rhodopsin/GRK1 interaction in the Tango assay (left panel) and in the AlphaScreen assay (right panel). Statistics to the DMSO control. **(B)** Mutations that disrupt the active conformation of the kinase domain abrogate rhodopsin/GRK1 interaction in the Tango assay. Mutations of human K219 and E238 equal bovine K216 and E235 as shown in the structure, respectively. Statistics were referred to the WT GRK1. **(C)** Examination of RH domain/kinase domain interface mutations on receptor interaction by Tango assay. Mutations of human T212, Y255, R461 and E521 equal bovine T209, Y252, R458 and E518 as shown in the structure, respectively. Statistics were referred to the WT GRK1. **(D)** HDX perturbation heat map of rhodopsin-GRK1 versus apo GRK1 overlaid on the structure of GRK1 (PDB ID: 3C4W). The colors indicate the % difference in GRK1 deuterium exchange (color code at bottom) upon complex formation with rhodopsin. ^*^P < 0.05; ^**^P < 0.01; ^***^P < 0.001. n.s., not significant (differences relative to DMSO control (panel **A**) or FL GRK1 (all other panels)).

Although the intact RH domain (the core domain fused to the C-terminal extension, 1-183 + 508-559) has full receptor interaction capacity in isolation, binding capacity may be modulated by the kinase activity in the context of the FL protein. Numerous studies have suggested that an active conformation of the kinase domain, characterized by four correctly aligned key hydrophobic residues (the “regulatory spine”), is needed for both receptor binding and phosphorylation, and conversely that receptor binding stimulates GRK kinase activity^7^. To test whether an active kinase domain conformation is required for Rho binding in the context of FL GRK1, we included the ATP-competitive GRK inhibitor paroxetine into our Tango assay. ATP competitive inhibitors mimic the effect of ATP in stabilizing an active kinase conformation, but block catalytic activity because they cannot be hydrolyzed^30^. As shown in Figure 3A, paroxetine indeed increased the interaction between receptor and GRK1, similar to the ATR ligand. Similar results were also obtained in an AlphaScreen assay, in which paroxetine increased the interaction between Rho and GRK1 *in vitro*. We next examined whether mutations of the critical ATP-binding residue K219 in β3 and of E238 in αC, which binds and positions K219 (nomenclature for human GRK1; shown as corresponding K216 and E235, respectively, in the bovine GRK1 structure in Figure 3B) (Figure 3B, left panel), affect the receptor binding capacity in our Tango system. Our data show that both K219A and E238A mutations almost completely eliminate the receptor binding capability of GRK1 (Figure 3B, right panel). We also tested the K219R mutant, a very conservative mutation that results in catalytic ally inactive protein, for which corresponding mutations do not affect folding in other protein kinases^31^. The K219R mutant also showed a complete block of receptor interaction. In contrast, K220 and D192 (shown as the corresponding K217 and D189 residues in the bovine GRK1 structure in Supplementary information, Figure S5A) form a comparably strong salt bridge within the relatively stable -sheet of the N-lobe, and their mutations only moderately affected receptor binding (Supplementary information, Figure S5A). Finally, we introduced triple P-to-A mutations into the PxxP motif (PPFKP) of the AGC C-tail in GRK1 (3PA, P470A/P471A/P474A; shown as corresponding P467, P468 and P471 in the bovine GRK1 structure in Supplementary information, Figure S5B), which have been shown to have the ability to disrupt receptor interaction and phosphorylation^32^. Consistent with a reported pull-down assay, the 3PA mutant shows no receptor interaction capacity (Supplementary information, Figure S5B), further indicating that an active kinase conformation is required for a stable interaction between GRK1 and Rho.

Since the kinase catalytic center is far from the RH domain, we speculated that the local conformational change caused by these mutations is transmittable to the RH domain through the interfaces that connect the kinase domain to the RH domain. There are two major interfaces between the RH domain and the kinase domain. One is the “upper” interface between the terminal subdomain (α1-3, α8-10) of the RH domain and the N-lobe of the kinase domain, and the other is the “lower” interface between the bundle subdomain (α4-7) of the RH domain and the C-lobe of the kinase domain (Figure 3C, middle panel). We then examined the effects of mutations of these interfaces. T212, Y255 and E521 (shown as corresponding to T209, Y252 and E518 in the bovine GRK1 structure in Figure 3C) on the upper surface form a hydrogen bond network that connects the α10 of the RH domain and the β-sheet bundle of the N-lobe. Mutations of these residues decreased receptor binding ability, and for the T212G mutation, in particular, almost totally abolished receptor interaction (Figure 3C, left lower panel). Similarly, F505 and F509 (shown as corresponding to F502 and F506 in the bovine GRK1 structure in Supplementary information, Figure S5C) form hydrophobic interactions with β4, β5 and αB of the N-lobe to hold the last helix of the kinase domain in position to connect with α10 of the RH domain. Mutations of those residues decreased receptor interaction (Supplementary information, Figure S5C). On the other hand, mutations of lower interface residues T97 and R461 (shown as corresponding to T97 and R458 in the bovine GRK1 structure in Figure 3C) did not affect receptor binding (Figure 3C, right panel), suggesting that a tight connection between the RH bundle domain and the C-lobe of the kinase domain is not needed for receptor binding.

We then used hydrogen-deuterium exchange mass spectrometry (HDX) to probe possible conformational changes of human GRK1 upon receptor binding. We first used HDX to study the dynamics of apo GRK1. The most stable regions we observed in apo GRK1 are the lower interface between the RH domain and the C-lobe of the kinase domain, as well as α9 and α10 of the upper bundle of the RH domain (Supplementary information, Figure S6A-S6B). Since the Rho/GRK1 interaction is quite weak and dynamic, we covalently fused Rho and GRK1 through a flexible linker to stabilize the complex to allow us to study potential conformational changes of GRK1 upon receptor binding. The biggest change we observed is the destabilization of the lower connection between the RH domain and the C-lobe of the kinase domain, indicating a possible dissociation of the connection upon receptor binding (Figure 3D, Supplementary information, Figure S6C). Although the magnitudes of differences are small, these are statistically significant and detected with confidence^33^. We also observed stabilization of α9 of the RH domain, while α10 of the RH domain seems to become more dynamic, suggesting an exquisite conformational change in this region upon receptor binding.

### Identification of key architecture elements crucial for receptor/kinase interaction

**Figure 4:**
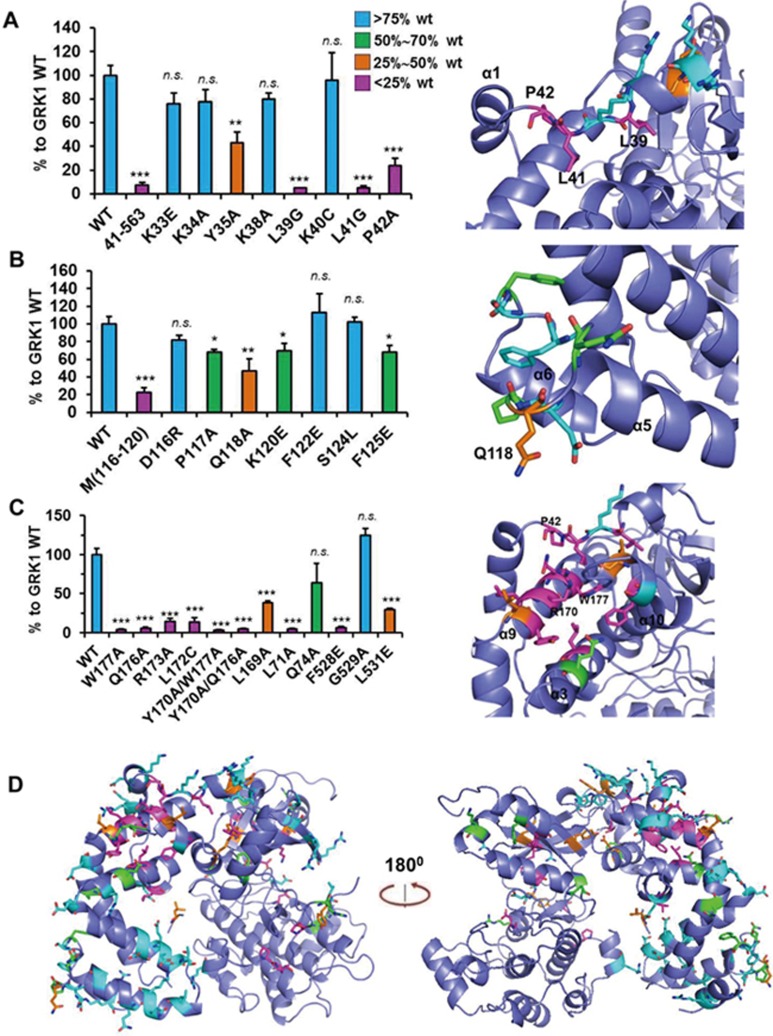
Identification of surface mutations of GRK1 crucial for rhodopsin binding. **(A)** Mutational analysis of residues in the loop between αNT and α1 of the RH domain for rhodopsin interaction via Tango assay. **(B)** Mutational analysis of residues in the α5-α6 loop of the RH domain. M(116-120): D116R/P117A/Q118A/K120E. **(C)** Identification of a cluster of mutations in α9, α3 and α10 of the RH domain important for receptor interaction via Tango assay. **(D)** An overview of all mutations overlaid on the structure of bovine GRK1 (PDB ID: 3C4W). ^*^P < 0.05; ^**^P < 0.01; ^***^P < 0.001. NS, not significant (differences relative to WT GRK1).

We next asked whether we can use the Tango assay to identify a surface on the RH domain that is crucial for receptor binding. We therefore performed an extensive mutational screen of the RH domain of GRK1. For easy comparison of data from different experimental batches, we normalized each mutation's activity to percent of WT GRK1 activity when ATR is present. In addition, we used a spectrum of colors, from bright blue to green, orange and magenta to visualize the severity of mutations for receptor binding, from slight (> 75% of WT), to medium (50%-75% of WT), severe (25%-50% of WT) and most severe (< 25% of WT), respectively, on the structure of GRK1 (PDB: 3C4W). This allowed us to easily identify a cluster of residues that is important for receptor binding. Our early domain mapping study showed that the first 30 amino acids are dispensable for receptor interaction, while further deletion of the N-terminus to residue 41 resulted in a complete loss of receptor binding (Figure 4A), suggesting that residues between amino acids 30 and 41 (the loop between αNT and the 1 helix of the RH domain) are important for receptor binding. We therefore mutated these residues one by one. The Tango data show that while mutations of the charged residues, K33E, K34A and K38A, failed to inhibit receptor binding, mutations of the hydrophobic residues L39, L41 and P42 severely affected receptor interaction.

We then analyzed the lower bundle of α4, α5 and α6 of the RH domain, which has been implicated in Gα binding in the structure of the GRK2/Gαq/Gβγ complex^34^. While a cluster of mutations of residues of the 5-α6 loop (D116R/P117A/Q118A/K120E) shows severe inhibition of receptor binding, single mutations only mildly affected receptor binding (Figure 4B). Similarly, a cluster of alanine mutations (L102A/Q105A/K106A/Q108A) in the lower helix bundle of the RH domain, as well as two clusters of mutations of charged residues, K133E/K135E and D91A/E93A/D94A/D96A, also failed to inhibit receptor binding (Supplementary information, Figure S7). Notably, the negatively charged cluster D91/E93/D94/D96 is highly conserved among all members of the GRK family. These data demonstrate that the lower bundle subdomain (α4-6) of GRK1 is not crucial for Rho binding, while the upper portion of the RH domain is required for Rho binding.

A study of sequence conservation across all members of the GRK family and the RGS proteins found that several residues in α9 and α3 of the RH domain are crucial for phosphorylation of the Rho C-terminus^35^. We tested two representative mutations, α9 Q176A and W177A, in our Tango system. Both mutations, either individually or combined with other mutations, completely eliminated receptor binding (Figure 4C). On the basis of this result, we performed an extensive mutational study of this region, which showed that most mutations in this region abrogated receptor interaction. To gain an overall view of the distribution and effect of all mutations (> 80 residues) that we have screened, we plotted mutated residues color coded for the strength of binding as percent of WT activity (Supplementary information, Table S1) on the structure of GRK1 (Figure 4D). While most mutations of the RH domain residues had little effect on receptor binding, we identified a cluster of mutations on α9, α3 and α10 that strongly repressed receptor binding (Figure 4D and 4C, right panel). Interestingly, these mutations are adjacent to the P42 and L41 mutations in the loop connecting αNT with RGS α1 (Figure 4C and 4A, left panel), which together form a surface groove encompassing α9, α3, α10 and the αNT-α1 loop. Unlike this cluster, all other mutations that severely inhibited receptor binding, including mutations in the kinase domain, were distributed rather sporadically (Figure 4D), suggesting that this surface groove is crucial for receptor binding.

**Figure 5:**
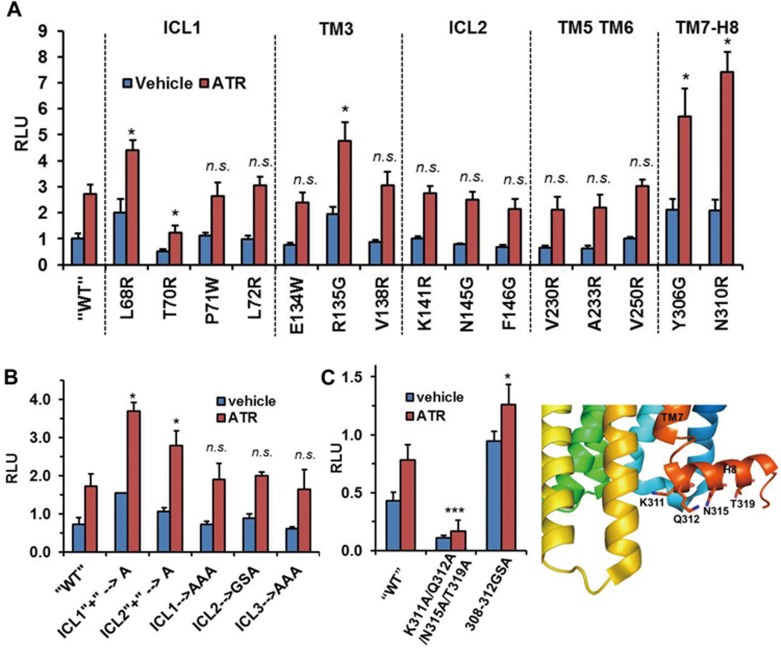
Identification of human rhodopsin (1-321) mutations that compromise the rhodopsin-GRK1 interaction in the Tango assay. **(A)** Screen of single mutations in different regions of rhodopsin for GRK1 interaction in the Tango assay. **(B)** Cluster mutations in the ICL of rhodopsin show no effect on GRK1 interaction in the Tango assay. ICL1“+”→A: H65A/K66A/K67A/R69A; ICL2“+”→A: K141A/R147A; ICL1→AAA: T62A/V63A/Q64A; ICL2→GSA: K141G/P142S/M143A/N145A/F146G/R147A/F148G/G149S; ICL3→AAA: Q236A/Q237A/Q238A. **(C)** A cluster of mutations in helix 8 of rhodopsin severely inhibit GRK1 interaction in the Tango assay. 308-312GSA: M308G/M309S/N310A/K311G/Q312A. Mutations are overlaid on the structure of bovine rhodopsin (PDB ID: 2X72). “WT” means rhodopsin 4M (1-321), all other mutations are based on this backbone. ^*^P < 0.05; ^**^P < 0.01; ^***^P < 0.001. ICL, intracellular loops; NS, not significant (differences relative to “WT” rhodopsin).

We then asked whether we could use a similar strategy to identify a region on the receptor side that is crucial for GRK1 interaction. We first performed a series of single mutation screens as we did in our previous study of the Rho/arrestin interaction. Surprisingly, except T70R of ICL1, most single mutations in Rho did not markedly decrease the GRK1 interaction signal and a number of single mutations, including L68R, R135G, Y306G and N310R, showed increased interaction with GRK1 (Figure 5A). Since a number of studies suggested that the intracellular loops of GPCRs may mediate the interaction with GRKs^12^, we introduced clusters of mutations to change the nature of the loop regions. However, these mutations did not decrease GRK1 interaction (Figure 5B). In addition, TM7 and helix 8 have been shown to play a crucial role in the recognition of arrestin. We therefore asked whether this region contributes to GRK1 interaction. While the kink mutation (308-312 GSA, M308G/M309S/N310A/K311G/Q312A) failed to repress the GRK1 interaction, a cluster of mutations on the cytoplasmic surface of helix 8 (K311A/Q312A/N315A/T319A) strongly inhibited the GRK1 interaction (Figure 5C), suggesting that helix 8 is important for this interaction.

### Mechanism of the gain-of-function mutation Q41L of GRK5 in enhancing receptor desensitization

**Figure 6:**
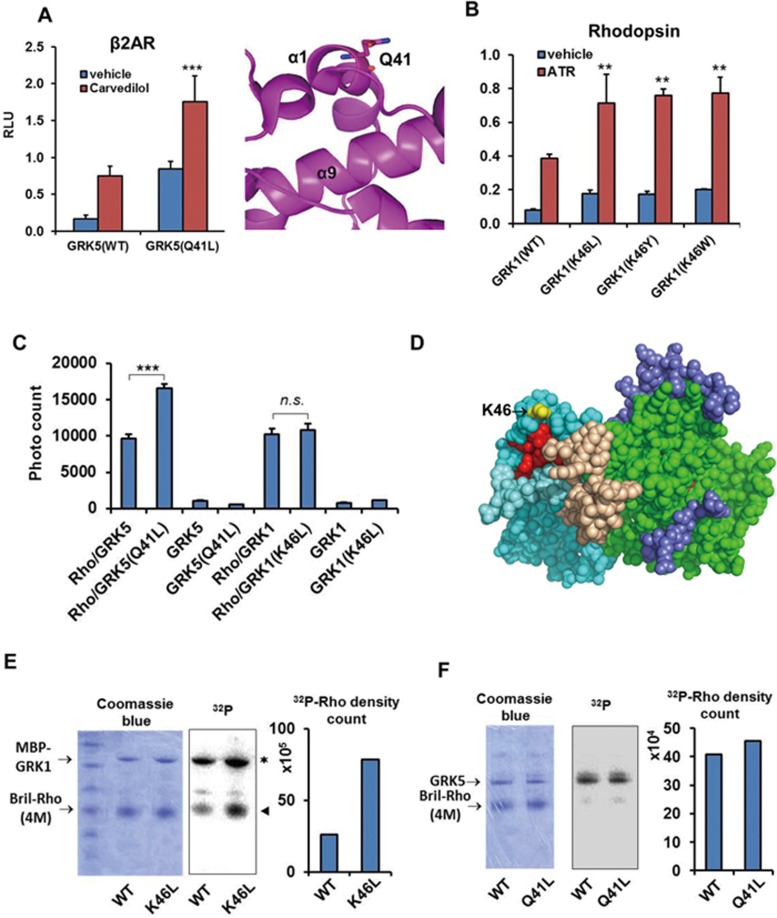
Mechanism of the gain-of-function Q41L mutation of GRK5 in receptor desensitization. **(A)** The Q41L mutation strongly increases the GRK5/β2AR interaction in the Tango assay. **(B)** Substitution of the corresponding residue in GRK1(k46) with hydrophobic residues increases the rhodopsin interaction. **(C)** Q41L increases the GRK5/rhodopsin interaction in the AlphaScreen assay. **(D)** K46 lies on the upper surface of the RH domain of bovine GRK1 (PDB ID: 3C4W). Yellow: K46; red: mutations of α3, α9 and α10 that severely decrease rhodopsin binding; cyan: RH domain (light cyan: RH α10); light brown: αNT; green: kinase domain; blue: C-tail of the kinase domain. **(E)** Direct kinase assay of the K46L of GRK1. **(F)** Direct kinase assay of the Q41L of GRK5. ^*^P < 0.05; ^**^P < 0.01; ^***^P < 0.001. NS, not significant (differences relative to WT GRK1).

Heart failure is a lethal disease that is in part caused by hyper activation of β-adrenergic receptors. A genetic study discovered that Q41L, a GRK5 polymorphism commonly found in African-Americans, has a protective effect against heart failure, acting like a “genetic β-blocker” to protect against death or cardiac transplantation^10^. This study showed that the Q41L mutation of GRK5 augments β2AR desensitization and provided evidence of diminished downstream receptor signaling, e.g., decreased adenylyl cyclase activities. However, the exact mechanism by which the Q41L mutation enhances the desensitization of receptor signaling remains unclear. The Q41 residue resides on the helix 1 of the RH domain of GRK5^36^ (Figure 6A, right panel). The Q41-corresponding residue in GRK1, K46 (Figure 6D, yellow sphere) is on the surface directly adjacent to the residues of the contiguous hydrophobic surface (Figure 6E, red spheres) that are required for receptor binding, suggesting the Q41L mutation of GRK5 might enhance receptor interaction by extending the hydrophobic surface (Figure 6A, right panel). Employing the Tango assay to interrogate the interaction between β2AR and GRK5, our data show that the Q41L mutation of GRK5 indeed strongly increased the interaction (Figure 6A, left panel). Since our Tango assay suggested that different GRKs may use a similar mechanism (e.g., utilize the RH domain) for receptor interaction (Supplementary information, Figure S4B and S4C), we asked whether the mutation corresponding to Q41L of GRK1, K46L has a similar effect on the Rho interaction. Therefore, we mutated K46 to the hydrophobic residues L, Y or W. All three mutations increased the interaction of GRK1 with Rho (Figure 6B), suggesting that GRKs may use this region to contact the hydrophobic core of the receptor. Since our work was focused on Rho, we employed an AlphaScreen assay to test the effects of all these mutations, including both GRK1 K46L and GRK5 Q41L, on the physical interaction of GRK1 or GRK5 with Rho. As shown in Figure 6C, the Q41L mutation clearly increased the physical interaction between GRK5 and Rho, while K46L only had a marginal effect on the GRK1- Rho interaction. We also used the direct kinase assay to examine the effect of these mutations on phosphorylation of Rho. The data showed at similar amount of loading (Figure 6E, coomassie blue stain, left panel); GRK1 (K46L) slightly increased autophosphorylation, but strongly increased (approximately three-fold relative to WT GRK1) the phosphorylation level of rho (4M) substrate (Figure 6E, middle and right panel). On the other side, compared to GRK1, GRK5 only weakly phosphorylates the Rho substrate (Figure 6F). This may be because Rho is not the native substrate of GRK5. Although not as dramatic as the K46L mutation of GRK1, we still found that the Q41L of GRK5 slightly increased the phosphorylation of Rho to about 10% above the level of the WT GRK5. We noticed that the K46L has a much stronger effect on the activity of GRK1 in the Tango assay than in the AlphaScreen assay (Figure 6B and 6C). We reasoned that this may be due to the membrane environment difference and the fact that the AlphaScreen is more sensitive to the slight change of protein concentration *in vitro*. On the other side, the kinase assay is highly consistent with the Tango assay where K46L has a strong effect on the phosphorylation of Rho (Figure 6B and 6E). Taken together, these data show that the GRK1 K46L mutation strongly enhances the phosphorylation of Rho, in a similar way as Q41L of GRK5 to augment the phosphorylation of β2AR^37^. These discoveries provide a mechanistic rationale for the gain-of-function Q41L mutation for receptor desensitization.

### Electron microscopy reveals the overall architecture of the rhodopsin/GRK complex

**Figure 7:**
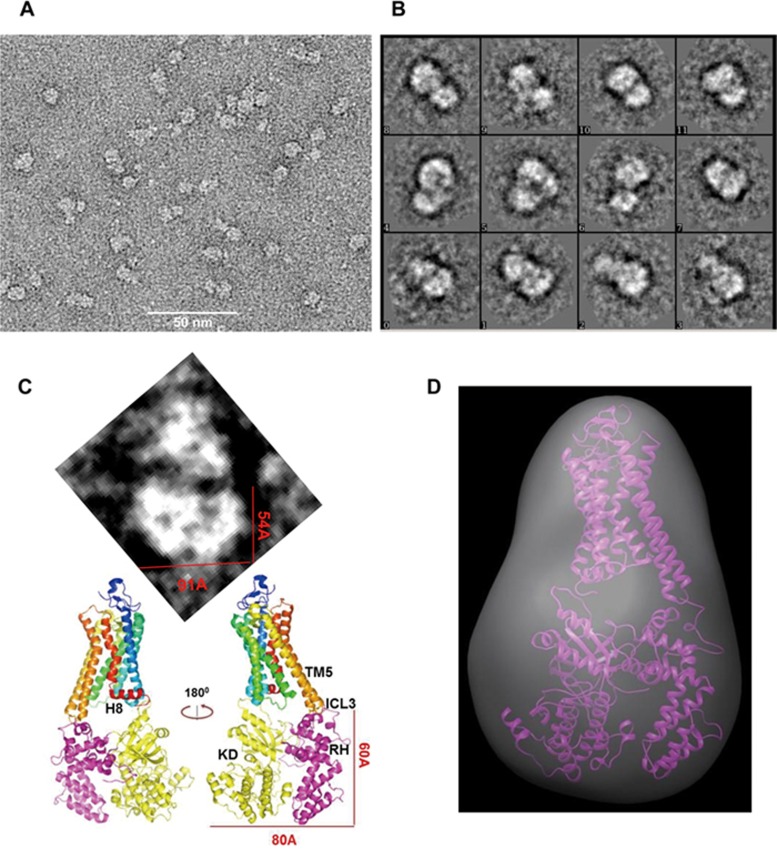
Negative stain EM reveals the principal architecture of the rhodopsin/GRK5 complex. **(A)** Representative EM image of negatively stained rhodopsin-GRK5 (Q41L) fusion protein in amphipol. **(B)** 2D classification of negative stain images. **(C)** 2D average of negative stain images (upper panel) and a proposed model of the rhodopsin/GRK5complex. **(D)** 3D reconstitution of the 2D negative stain images of the rhodopsin/GRK5 complex overlaid with modeled rhodopsin/GRK5 complex (rhodopsin: PDB ID: 3PQR; GRK5: PDB ID: 4TND). Refined resolution was at 23 Å.

The main challenge of gaining structural insight into the GPCR/GRK interaction is the weak and dynamic association between receptor and kinase. Since Q41L of GRK5 shows an enhancement of receptor interaction both in live cells and in the context of purified recombinant proteins, we asked whether we could use the Q41L mutation of GRK5 to probe the architecture of the Rho/GRK interaction. To further overcome the weak interaction between the receptor and the kinase, we covalently fused Rho to GRK5 (Q41L) via a flexible linker to acquire structural information of Rho/GRK. We have successfully used a fusion protein strategy to gain structural insight into many weakly associated protein complexes, such as the Jaz9/MYC3^38^, SnRK/PP2C^39^ and the Rho/arrestin complexes^15^. To test whether the receptor-fused kinase is active, we use the direct kinase assay to examine the kinase activity of the receptor-kinase chimera. Our data show that both the GRK1- and GRK5 (Q41L)-receptor fusion proteins can efficiently phosphorylate the chimera kinase itself (autophosphorylation) and the Rho substrate (Supplementary information, Figure S8A). We noticed that Rho is less efficiently phosphorylated in the context of the GRK5 chimera than the GRK1 chimera. This is consistent with our previous observation (Figure 6E and 6F) and with Rho not being the native substrate of GRK5. Single-particle cryo-EM is an emerging tool of current structural biology. Because of the dynamic nature of the GPCR protein and the size of our complex, obtaining high-resolution images of the Rho/GRK5 complex via cryo-EM is currently impractical. We therefore decided to use negative stain EM to shed light on the overall architecture of the Rho/GRK complex. We expressed and purified BRIL-Rho-GRK5 (Q41L) fusion protein as monodisperse protein in insect cells (Supplementary information, Figure S8B). EM images from negatively stained Rho/GRK5 complex in amphipols, a new class of stably binding surfactants, show that the receptor/kinase complex forms an average 14× 10 nm particle, roughly the same size as Rho and GRK5 stacked together (Figure 7A). 2D classification of 8 000 particles shows the majority of the complex contains two parts; the upper part has a conical shape that resembles Rho, while the slightly bigger lower part comprises two interconnected domains that resemble the shape of GRK5 (Figure 7B). Interestingly, we can also visualize the shape and the connection of the two domains of GRK5 from the 2D average of 1 100 particles (Figure 7C, upper panel), with the slightly smaller domain on the left resembling the RH domain and the slightly bigger on the right resembling the kinase domain of GRK5. GRK5 packs against Rho through the upper parts of both the RH domain and the kinase domain. On the basis of the outward movement of TM5/TM6 in the active conformation of Rho, it is conceivable that Rho uses the outstretched ICL3 loop to contact the RH domain of GRK5 and helix 8 or ICL1 to contact the kinase domain (Figure 7C, lower panel). However, because of the low resolution of the negative stain images, we cannot rule out the alternative orientation, in which the kinase domain packs against ICL3 and the RH domain contacts helix 8 or ICL1. A 3D reconstitution of the 2 000 2D images suggests that the upper part of the RH domain contacts the loop region of Rho (Figure 7D). Notably, this interface includes the surface required for Rho binding and Q41. It is therefore reasonable that the Q41L-mutated residue of GRK5 may directly contact ICL3 or the TM5-TM6 core. Taken together, the low-resolution EM images of the Rho/GRK5 complex reveal the main architecture of the Rho/GRK interaction in which GRK5 utilizes both the RH domain and the kinase domain to interact with the intracellular part (including both ICL3 and helix 8) of Rho.

## Discussion

GPCR signaling is mainly mediated by G proteins, arrestins and GRKs. The structural information of these mediators in complex with receptor is crucial for understanding GPCR signaling, and possesses great translational potential for a broad spectrum of diseases. Compared to GPCR/G-protein and GPCR/arrestin complexes, the structure determination of a GPCR/GRK complex is even more challenging due to the weak and transient interaction between receptor and GRK. Some of the most important questions about the GPCR/GRK interaction are: (1) which domain of GRK mediates receptor binding? (2) Is an active conformation of the kinase domain required for receptor binding? (3) What are the main interfaces and what are the key architectural elements of the receptor/kinase complex? We were able to address these important questions by combining a modified Tango system, which could effectively detect the weak and transient interaction between receptor and kinase, with different biochemical approaches and single-particle negative stain EM imaging.

The RH domain of GRK has long been suspected to play a crucial role in receptor recognition and binding, but its exact role remained unclear. We have used the Tango system to systematically analyze the Rho/GRK1 interaction and demonstrated that the RH domain is the main domain of GRK1 for receptor binding. One of the biggest conundrums of the GPCR/GRK interaction has been the role of the very N-terminus of GRKs. Numerous studies based on mutations, deletions, peptides and antibodies directed against this region have demonstrated its importance for receptor phosphorylation, and also suggested that this region is important for receptor interaction^28,29,40^. However, a study of the metabotropic glutamate receptor 1 showed that the RH core domain lacking the N-terminal region (GRK2(45-185)) still effectively binds to the receptor^41^, arguing that the very N-terminus is dispensable for receptor binding. Consistent with this observation, our deletion study showed that the RH core domain without the very N-terminus, GRK1 (31-183), retains most of the receptor binding capacity (∼70%). On the other side, our kinase assay confirms that the very N-terminus is essential for GRK1's kinase activity to phosphorylate Rho substrate, but not for its autophosphorylation activity. Structural studies of the GRK family have suggested that αNT is flexible and able to adopt different conformations. We therefore postulate that binding of GRK to receptor is a multiple-step process: the first step is that the RH domain of GRK recognizes the core domain of an activated receptor, and this recognition brings αNT of GRK into the constrain of receptor loop regions, which forces αNT to tilt toward the kinase domain and make contact with the N-lobe and the AGC C-tail to lock the kinase domain in the active conformation that is required for receptor phosphorylation. This explains why αNT is not important for receptor binding but is crucial for receptor phosphorylation. Although our study found that αNT is not absolutely necessary for receptor binding, we cannot rule out the possibility that αNT may somehow contribute to receptor binding as the flexible αNT may contact the loop region of the receptor when the receptor and kinase engage with each other.

Receptor activation is believed to be a precondition for GRK engagement, while numerous studies also suggested that an active conformation of the kinase domain is needed for receptor binding^26,42^. Our Tango data clearly show that GRK1 prefers the active conformation of Rho (Figure 1B), and our mutation and kinase inhibitor data also confirm that the active conformation of the kinase domain is required for receptor binding. Together with the data of the RH domain as main receptor interaction domain, our study suggests that in the context of FL GRK, receptor binding may involve both the kinase domain and the RH domain in a collaborative way. More importantly, the hypothesis is supported by our negative stain EM data, which clearly show that both the RH domain and the kinase domain can make contacts with the intracellular part of the receptor.

A key discovery of our study is the identification of an RH domain surface that encompasses α3, α9, α10 and the αNT-α1 loop as the key component that is critical for receptor interaction. This key surface may not completely contact the receptor, but may be essential to support a conformation required for receptor binding. Our negative stain EM data support this observation as one of the major receptor interfaces is formed by the surface that we identified in our Tango mutation screen. On the other side, we identified helix 8 of Rho as being important for GRK1 binding. This discovery is also supported by our EM data as the image reconstruction shows that the upper surface of the kinase domain contacts helix 8 and probably also ICL1. Collectively, the EM reconstruction supports the interface identified by the Tango assay mutant screen.

The gain-of-function mutation Q41L of GRK5 is of particular interest, not only because this mutation has a protective effect against heart failure, which may have a further therapeutic implication^10^, but also because this mutation is crucial for understanding the mechanism by which GRKs recognize and interact with GPCRs. The Q41 residue lies on the surface of helix 1 of the RH domain, which is a potential surface to directly interact with the receptor. Our Tango assay and *in vitro* AlphaScreen assay clearly demonstrate that the Q41L mutation has the ability to increase receptor binding, arguing that this region might be the receptor interface. This is consistent with our early Tango mutation screen data in this region as these data show that deletion of this region (residues 30-41) abolishes receptor binding, and mutations of hydrophobic residues in this region severely impair receptor binding (Figure 4A). Further support of the importance of the interface for receptor binding comes from the fact that the Q41L mutation is adjacent to the hydrophobic surface groove required for receptor binding (Figure 6A, 6D). The final support of the interface comes from the EM negative stain images, which show that GRK5 indeed uses this surface to contact the intracellular region of Rho. Collectively, these data indicate that the protective effect of the Q41L mutation against heart failure is through enhancing the receptor interaction, and thus facilitating receptor phosphorylation, which leads to inhibition of downstream GPCR signaling. We hypothesize that the Q41L mutation may have a hydrophobic interaction with the hydrophobic core of the receptor, but current low-resolution EM images cannot provide further details. Future work on strengthening the interaction via both mutation and crosslinking could greatly increase the possibility of obtaining the complete structural information of the complex via cryo-EM.

In summary, we have discovered key components for the Rho/GRK1 interaction and revealed the main architecture of the Rho/GRK complex through combinational use of a genetic approach with biochemical methods, and single-particle EM analysis. Our work provides a framework for understanding the desensitization of GPCRs and generates insightful information for future high-resolution structure determination of a GPCR/GRK complex.

## Materials and Methods

### Tango assay

The Tango system was adapted from that of a previous report^15,21^. As membrane protein, a GPCR (human Rho, β2AR, PTH1R or HTR1b) was fused at the N-terminus of an optimized TEV cleavage site (ENLYFQS), followed by a modified transcriptional activator based on the Clontech Tet-ON 3G transactivator, which is more robust and specific than conventional tTA. The Tet-ON 3G transactivator is mutated at K71E and N95D to render it constitutively active without doxycycline binding. The whole-fusion construct is under the control of the CMV promoter in the pcDNA3 plasmid. For the cytoplasmic partner construct, a GRK (human GRK1, GRK5 or bovine GRK2)-TEV protease fusion was expressed under control of a CMV promoter on the pCDNA3 backbone. An hemagglutinin (HA) or FLAG tag was inserted between the GRK and the TEV protease genes for easy protein expression detection. HTL cells were a gift from G Barnea and R Axel (Brown University and Columbia University), and were cultured in DMEM medium with 10% FBS. Generally, 10 ng receptor construct and 10 ng GRK construct, together with 1 ng phRG-tk Renilla luciferase expression plasmid were transfected with Xtremgene 9 (Roche) according to the manufacturer's instruction. However, under conditions where the expression levels of mutant or deletion constructs were lower than WT, the amount of DNA transfected was adjusted to achieve similar protein expression levels based on western blot results of Supplementary information, Figures S2 and S3. One day after transfection, cells were induced by vehicle or ligand overnight. Cells were harvested and lysed in Passive Lysis Buffer (Promega). Luciferase activity was measured using the Dual Luciferase Kit (Promega) according to manufacturer's instructions.

### Protein expression and purification

We used human Rho and GRK1 if not specified otherwise. H8-MBP-Rho (4M) protein was expressed using a tetracycline-inducible expression cassette construct in HEK293S cells (Invitrogen) transiently transfected using Lipofectamine 2000 (Invitrogen) as described previously^15^. Biotin-MBP-GRK1 and Arrestin-biotin-MBP coding regions were subcloned into pFastBac-Duet vector (Invitrogen) and expressed in Sf9 insect cells. All MBP-tagged proteins were affinity purified on amylose beads as described before^15^. Biotin-MBP tag was removed by 3C protease digestion and Ni-NTA beads purification. For Rho-GRK5 fusion protein for EM analysis, thermally stabilized apocytochrome b_562_RIL^43^ was fused to the N-terminus of the FL Rho, and a 4× GSA linker was inserted between the C-terminus of Rho and the N-terminus of FL GRK5. Then the receptor chimera sequence was subcloned into a modified pFastBac1 vector (Invitrogen), which contained a cassette for expression of a HA signal sequence followed by a FLAG tag, a 10× His tag and a TEV protease recognition site at the N-terminus of the receptor sequence. The resulting receptor chimera construct was transformed into DH5-Bac competent cells to generate bacmid, and subsequently transfected into Sf9 cells to generate baculovirus according to the manual of Bac-to-Bac Baculovirus Expression System (Invitrogen). The fusion protein was purified using Ni-NTA beads as described above.

### AlphaScreen for detecting protein interactions *in vitro*

Interactions between human Rho and human GRK were assessed by a luminescence-based AlphaScreen assay (Perkin Elmer). The AlphaScreen principle is illustrated in Figure 1C. Briefly, biotinylated GRK1 was bound to streptavidin-coated donor beads and His8-tagged Rho was bound to nickel-chelated acceptor beads. The donor and acceptor beads were brought into close proximity by the interactions between Rho and GRK. When excited by a laser beam of 680 nm, the donor beads emit singlet oxygen that activates thioxene derivatives in the acceptor beads, which releases photons of 520-620 nm as the binding signal. The experiments were conducted with 40 nM of Rho and GRK proteins in the presence of 5 μg/ml donor and acceptor beads in a buffer of 50 mM MOPS-Na, pH 7.4, 50 mM NaF, 50 mM CHAPS, 0.01% DDM and 0.1 mg/ml bovine serum albumin. The results were based on an average of three experiments with SE typically < 10%.

### GRK1 kinase activity determination by AlphaScreen

FL and fragments of human GRK1 protein were expressed in Sf9 cells and purified as described under the protein expression and purification section. The kinase activity was examined via AlphaScreen assay for interaction between human WT arrestin (biotinylated) and human 4M Rho (His8 tagged). Specifically, 80 nM purified GRK1 protein was added to the arrestin/Rho (4M) interaction mixture as described under AlphaScreen assay. ATP was then added to a final concentration of 40 μM. The kinase activity was measured as photo count of the interaction between WT arrestin and 4M Rho.

### Direct kinase assay via radioactive ^32^P-γ-ATP

The expression and purification of GRK and Rho protein were described in the protein expression and purification section. For the kinase assay, 2 μg of purified BRIL-Rho (4M) was mixed with 0.5 μg of purified GRK protein (MBP-GRK1 or GRK5) in 20 μl of kinase reaction buffer (20 mM Tris-Cl, pH 7.5; 10 mM MgCl_2_; 1 mM DTT; 250 μM ATP), 0.2 μl ^32^P-γ-ATP (10 mCi/ml, 3 000 Ci/mmol) and incubated for 30 min at room temperature. The kinase reaction was stopped by adding equal volume of 2× SDS loading buffer. Then samples were loaded onto SDS-PAGE gels. After electrophoretic separation, gels were first stained with Coomassie Blue to visualize the protein, then the gels were dried and scanned by PhosphorImager. Densitometry was performed by ImageQuan TL (GE Life Sciences).

### Hydrogen-deuterium exchange mass spectrometry

HDX of human GRK1 was carried out as described previously^15^, with the following modifications: (1) the solution handling and mixing was performed with a LEAP Technologies Twin HTS PAL liquid handling robot housed inside a temperature-controlled cabinet held at 4 °C^44^ and decyl maltose neopentyl glycol was used in place of DDM in the exchange buffer. Digestion was performed in line with chromatography using an in-house packed pepsin column. Peptides were captured and desalted on a C8 trap. Peptides were then separated across a 5 μ 10×1 mm Betasil C8 column (Thermo Fisher Scientific) with a linear gradient of 12-40% acetonitrile in 0.3% formic acid over a short 5 min gradient to limit back exchange with the solvent. Mass spectra were acquired in the range of *m/z* 300-2 000 at a resolution of 60 000 for 8 min in positive ion mode on a Q Exactive mass spectrometer (Thermo Fisher Scientific) equipped with an ESI source operated at a capillary temperature of 225 °C and spray voltage of 3.5 kV. Data were processed as described before^15^.

### Electron microscopy

For negative staining, the human BRIL-Rho-GRK5 fusion in amphipols or detergent micelles was stained by conventional uranyl formate negative staining. The negative-stained sample was imaged at room temperature with a Tecnai G^2^ Spirit electron microscope (FEI) at Van Andel Institute operated at 120 kV using low-dose procedures. Images were recorded at a magnification of 49 000× and a defocus value of 1.2 μm on an Eagle CCD camera. All images were binned (4 096 pixels) to obtain a pixel size of 2.14 A° on the specimen level. 2D classification was performed by EMAN 2.1 or RELION 1.4 from 4 000 of handpicked or 8 000 automatically picked particles. 3D reconstitution was performed by EMAN2 or RELION 1.4 with 2 800 particles. To avoid bias, the 3D model was first *de novo* built by EMAN2^45^ without any structural clue, then RELION^46^ was used to refine the initial model.

### Antibodies and western blotting

To examine protein expression levels, Tango constructs were transfected into AD293 cells (Stratagene) in 24 wells at 80% confluency after 1 day of growth. Generally, 100 ng GRK-TEV fusion construct or receptor-tTA fusion construct were transfected into AD293 cells per well. Two days after transfection, cells were lysed by CelLytic M (Sigma) reagent according to the manufacturer's instructions. For western blotting, equal amounts of total protein lysates were loaded and separated by 4-20% gradient SDS-PAGE, followed by protein transfer to nitrocellulose membranes. Membranes were blocked with 10% milk and then incubated with the appropriate primary and secondary antibodies with extensive washes (three times) between each step. Chemiluminescence signals were detected by SuperSignal West Pico (Pierce). For the titration experiments, the WT GRK1 construct was transfected at a fixed dose of 100 ng per well (1×), and mutation or deletion constructs were transfected at various doses, and their protein expression levels were compared by western blotting. The levels of the Rho-tTA fusion proteins were detected by TetR monoclonal antibody from Clontech, and GRK-TEV fusion proteins were detected by anti-Flag M2 monoclonal antibody from Sigma.

### Statistical analysis

All reporter assays, AlphaScreen assays and kinase assays, were repeated at least 3 times with triplicate samples each time. Error bars indicate SD Data were analyzed by two-tailed, unpaired *t*-tests with GraphPad Prism 5 or Excel software. For statistics, ^*^P < 0.05; ^**^P < 0.01; ^***^P < 0.001, NS, not significant.

## Author Contributions

Conception: YH, KM, OPE and HEX; methodology: YH, XG, DG, LH, KP and YY; resources: GZ, PG, KM and HEX; writing: YH, KM and HEX. Funding acquisition: HEX, KM, PG and OPE.

## Competing Financial Interests

The authors declare no competing financial interests.

## Supplementary Information

Supplementary information, Figure S1 Replotting of domain mapping results as percentage of wildtype GRK1 interaction capacity reveals that the RH domain is the main receptor (Rho 1-321) interaction domain.

Supplementary information, Figure S2 Titration experiments for the Tango assay.

Supplementary information, Figure S3 Protein expression levels of key fragments/mutants of GRK or rhodopsin.

Supplementary information, Figure S4 Extended GRK1/receptor interaction data.

Supplementary information, Figure S5 Extended Tango data of kinase domain mutations on receptor interaction.

Supplementary information, Figure S6 HDX analysis of human GRK1 in the absence and presence of rhodopsin.

Supplementary information, Figure S7 Mutations of residues of the α4-α6 bundle of the RH domain of GRK1 do not inhibit rhodopsin binding in Tango assay.

Supplementary information, Figure S8 Biochemical properties of BRIL-rhodopsin-GRK5 fusion protein.

Supplementary information, Table S1 A summary of rhodopsin binding capacities of all GRK1 mutant proteins.
