# Supplementary material for: Molecular assembly of rhodopsin with G protein-coupled receptor kinases
Source: Cell Res. 2017 May 19;27(6):728–47. doi: 10.1038/cr.2017.72 (PMC5518878; doi:10.1038/cr.2017.72)
Supplement: Supplementary information, Figure S1 — Replotting of domain mapping results as percentage of wildtype GRK1 interaction capacity reveals that the RH domain is the main receptor (Rho 1-321) interaction domain. [file cr201772x1.pdf]

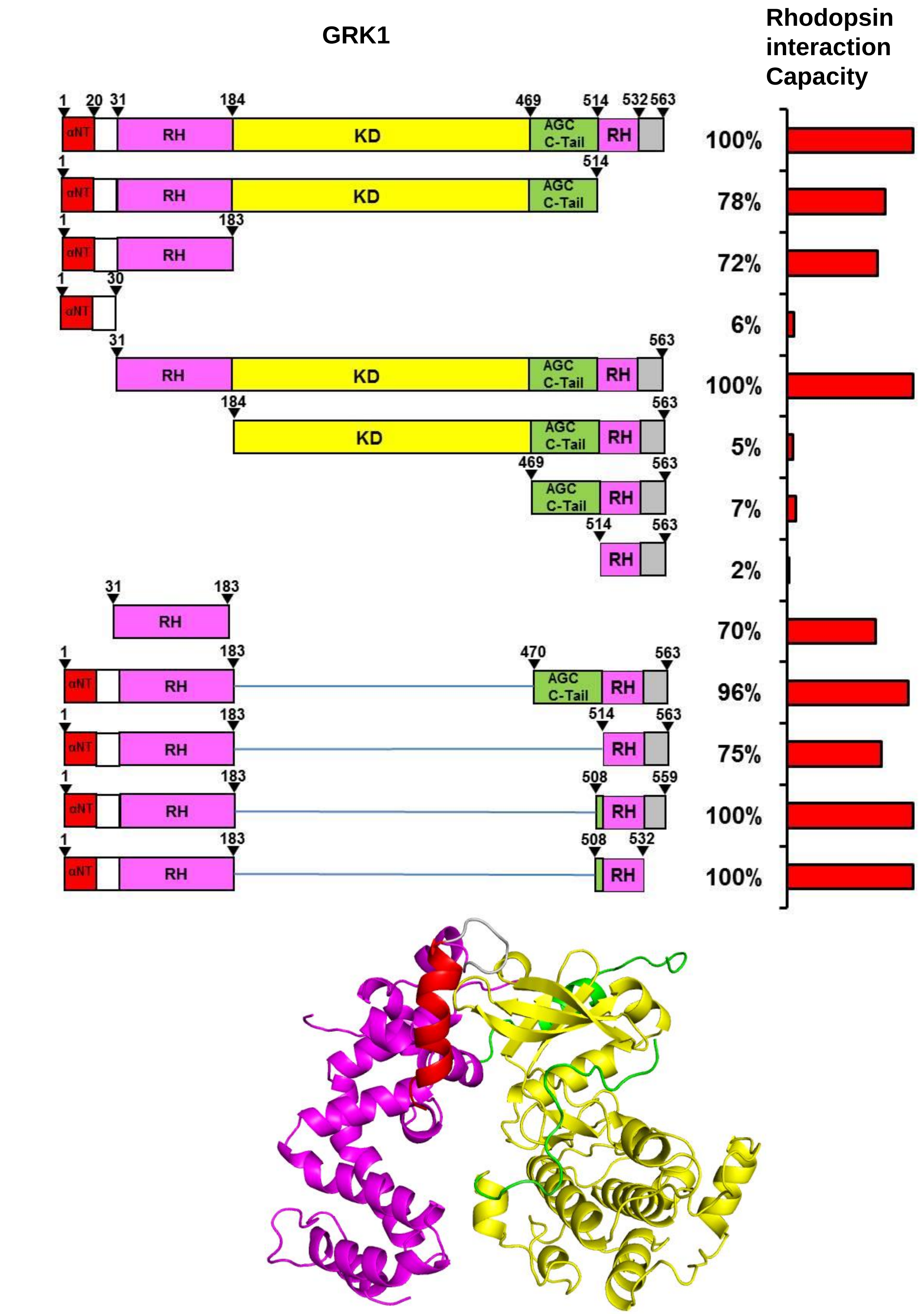


**Supplementary information, Figure S1.** Replotting of domain mapping results as percentage of wildtype GRK1 interaction capacity reveals that the RH domain is the main receptor (Rho 1-321) interaction domain. Representative structure is bovine GRK1, PDB ID, 3C4W.
