# Supplementary material for: Molecular assembly of rhodopsin with G protein-coupled receptor kinases
Source: Cell Res. 2017 May 19;27(6):728–47. doi: 10.1038/cr.2017.72 (PMC5518878; doi:10.1038/cr.2017.72)
Supplement: Supplementary information, Figure S2 — Titration experiments for the Tango assay. [file cr201772x2.pdf]

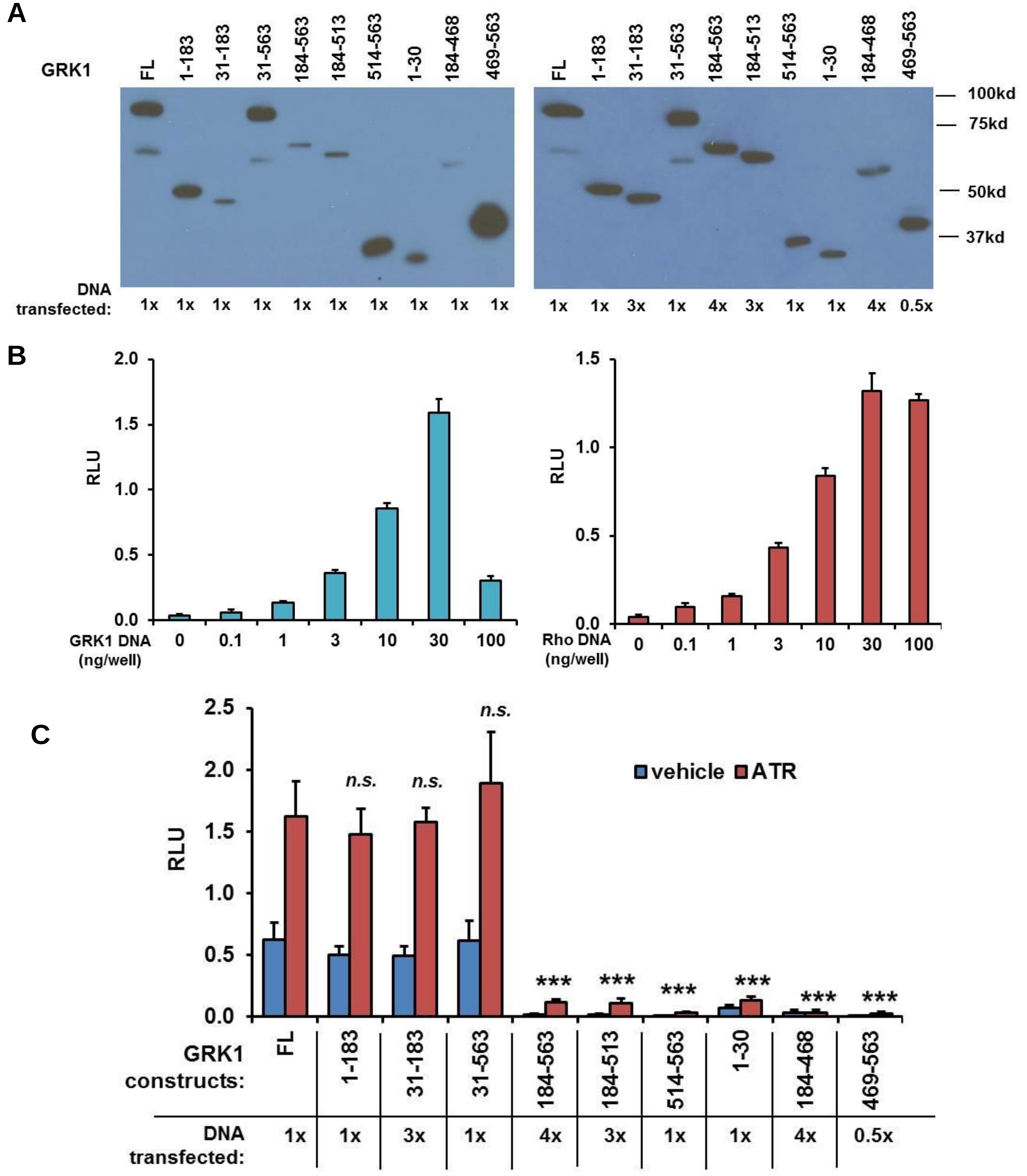

Supplementary information, Figure S2. Titration experiments for the Tango assay. (**A**) Protein expression titration experiment of GRK1 truncations. Protein levels of GRK1 deletion constructs at equal DNA amounts, left panel. Protein levels of GRK1 deletion constructs at titrated DNA amounts, right panel. The protein levels of GRK1-TEV fusion protein were detected by anti-FLAG antibody. (**B**) A dose response of the amount of DNA transfected in the Tango system. (**C**). Rhodopsin (1-321) interaction capacity of GRK1 deletion constructs at adjusted DNA amounts. *P<0.05; **P<0.01; ***P<0.001, n.s. not significant (differences relative to FL GRK1).
