# Supplementary material for: Molecular assembly of rhodopsin with G protein-coupled receptor kinases
Source: Cell Res. 2017 May 19;27(6):728–47. doi: 10.1038/cr.2017.72 (PMC5518878; doi:10.1038/cr.2017.72)
Supplement: Supplementary information, Figure S3 — Protein expression levels of key fragments/mutants of GRK or rhodopsin. [file cr201772x3.pdf]

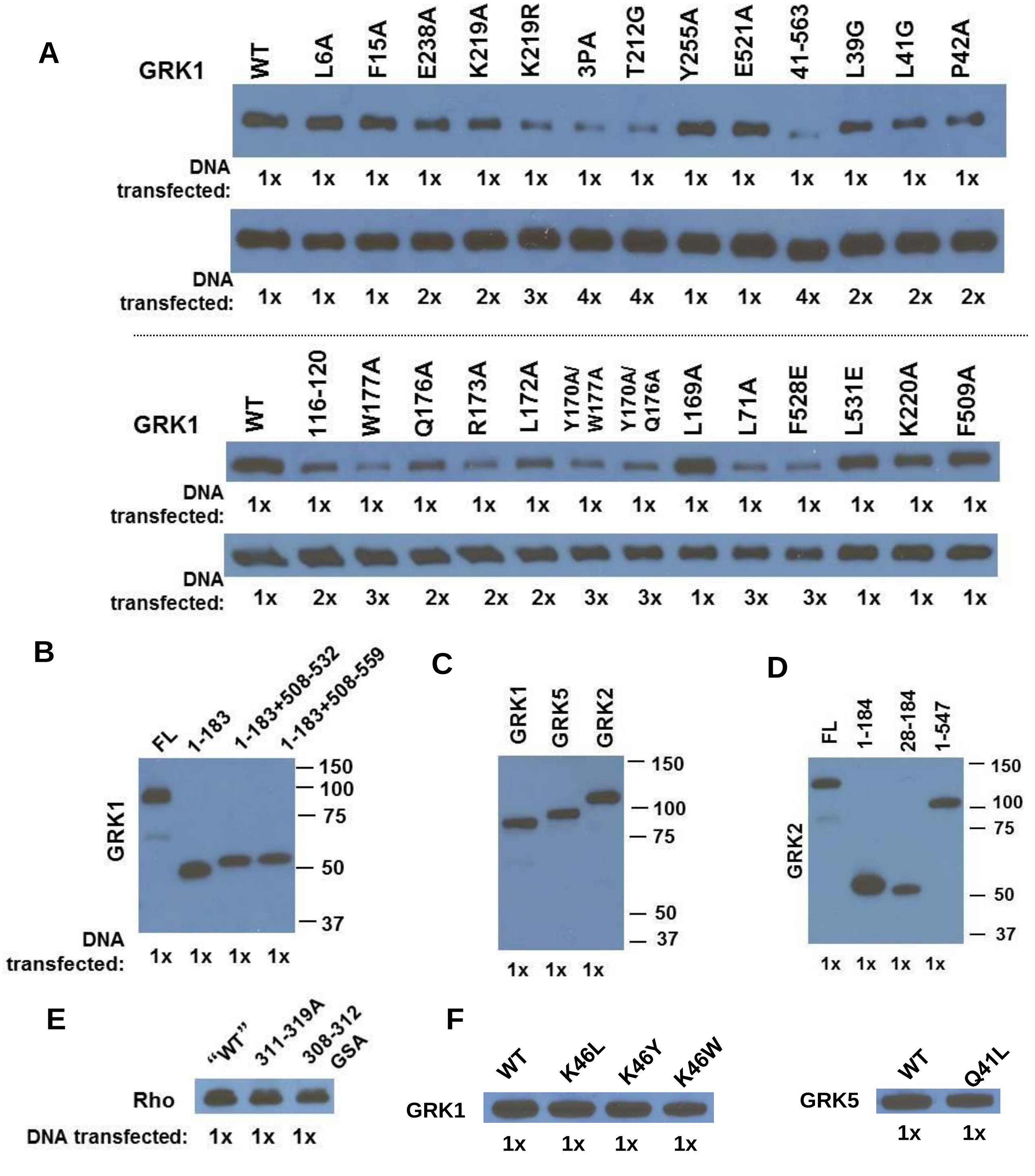

Supplementary information, Figure S3. Protein expression levels of key fragments/mutants of GRK or rhodopsin. (**A**) Protein expression levels of GRK1 key mutant proteins. The protein expression levels of GRK1-TEV fusion proteins were detected by anti-FLAG antibody. (**B**) Protein expression levels of RH domain constructs of GRK1. (**C**) Protein expression levels of GRK1, GRK5 and GRK2. (**D**) Protein expression levels of GRK2 deletion constructs. (**E**) Protein expression levels of rhodopsin TM7 and helix 8 key mutant proteins. 311-319A: K311A/Q312A/N315A/T319A; 308-312 GSA: M308G/M309S/N310A/K311G/Q312A.The protein expression levels of rhodopsin-tTA fusion proteins were detected by western blot probed with anti-TetR monoclonal antibody. (**F**) Protein expression of GRK1 K46 and GRK5 Q41 mutants, detected by anti-FLAG antibody.
