# Supplementary material for: Molecular assembly of rhodopsin with G protein-coupled receptor kinases
Source: Cell Res. 2017 May 19;27(6):728–47. doi: 10.1038/cr.2017.72 (PMC5518878; doi:10.1038/cr.2017.72)
Supplement: Supplementary information, Figure S4 — Extended GRK1/receptor interaction data. [file cr201772x4.pdf]

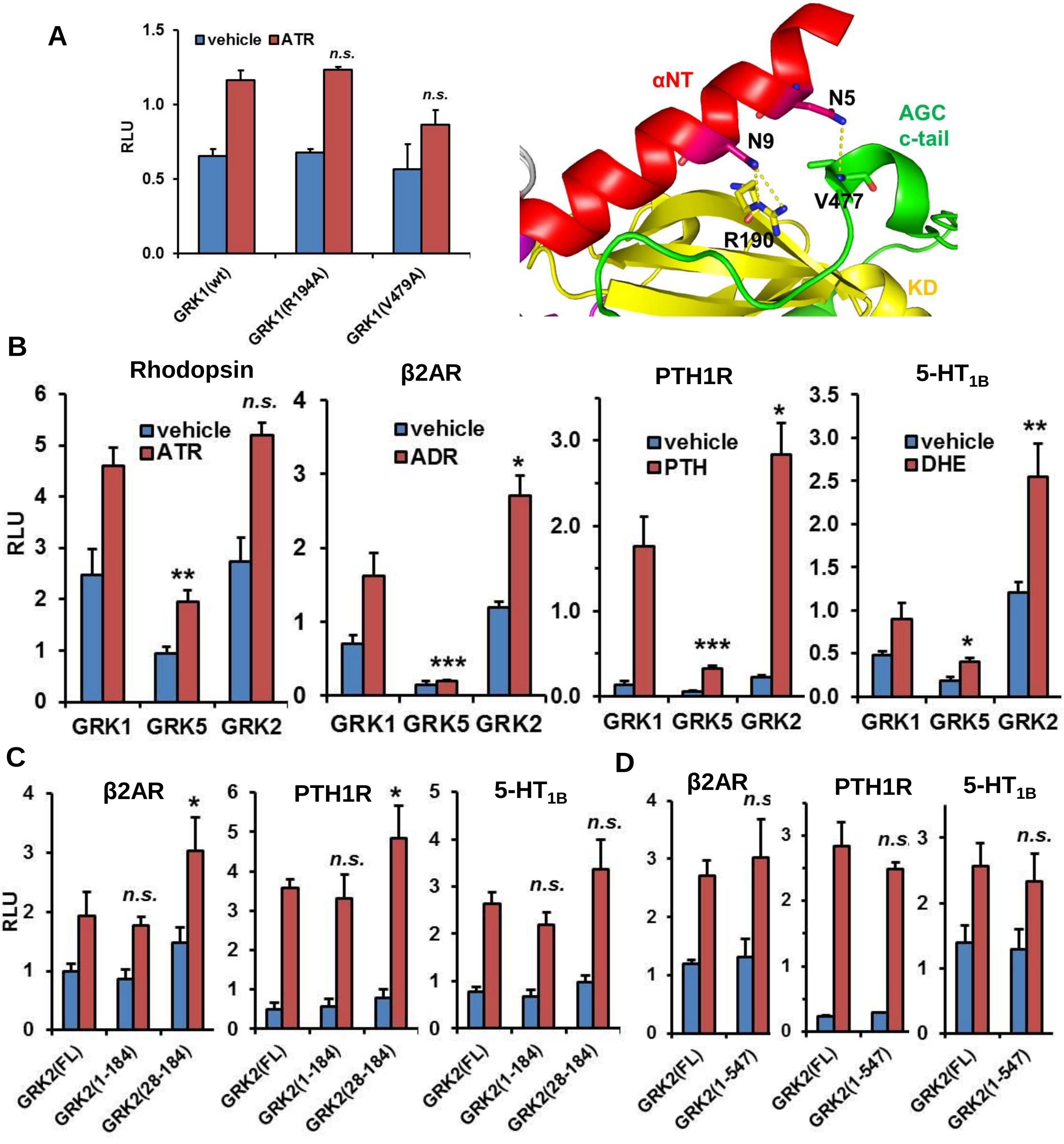

Supplementary information, Figure S4. Extended GRK1/receptor interaction data. (**A**) Mutations of αNT-interacting residues do not inhibit the GRK1/rhodopsin interaction in Tango assay. Representative structure, GRK6 (human), PDB ID: 3NYN. (**B**) Examination of the interactions of GRK1, GRK5 and GRK2 with rhodopsin, β2A, PTH1R and HTR1b in Tango assay. ATR, all-trans retinal, 10 µM; ADR, adrenaline, 10 µM; PTH, PTH(1-34), 0.5 µM; DHE, dihydroergotamine, 0.1 µM. (**C**) Examination of the N-terminal domain (RH core domain) of GRK2 for receptor interaction in Tango assay. (**D**) Examination of the PH domain truncation of GRK2 for receptor interaction in Tango assay. *P<0.05; **P<0.01; ***P<0.001, n.s. not significant (differences relative to WT GRK (panels A and B) or WT GRK2 (panels C and D).
