# Supplementary material for: Molecular assembly of rhodopsin with G protein-coupled receptor kinases
Source: Cell Res. 2017 May 19;27(6):728–47. doi: 10.1038/cr.2017.72 (PMC5518878; doi:10.1038/cr.2017.72)
Supplement: Supplementary information, Figure S5 — Extended Tango data of kinase domain mutations on receptor interaction. [file cr201772x5.pdf]

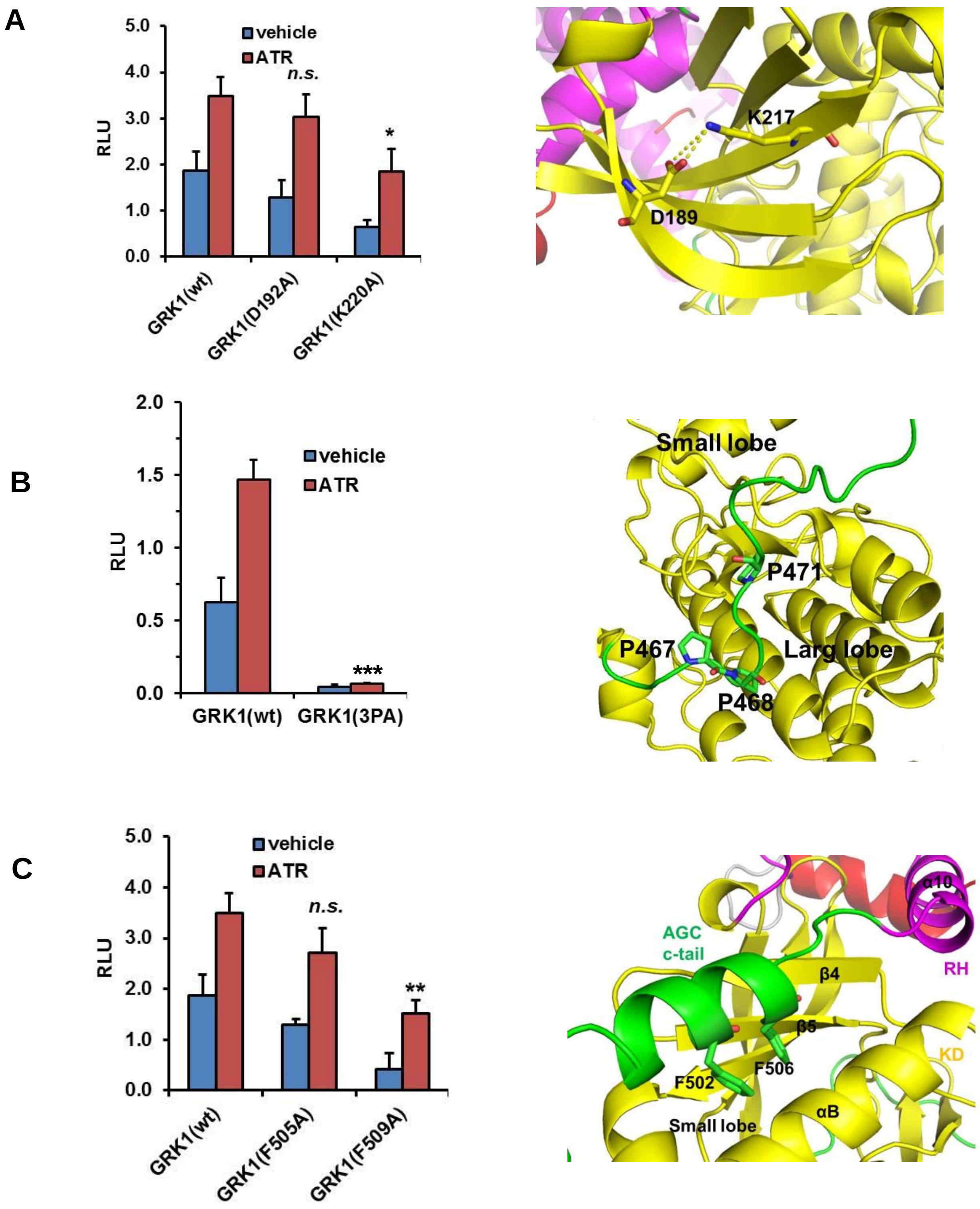

Supplementary information, Figure S5. Extended Tango data of kinase domain mutations on receptor interaction. (**A**) Mutation analysis of a salt bridge connection of the kinase domain for receptor interaction in Tango assay. (**B**) A triple proline-to-alanine mutation (3PA) in the AGC C-tail abolishes the rhodopsin/GRK interaction in Tango assay. (**C**) Mutations that affect the connection between the kinase domain and the RH domain decrease rhodopsin interaction. Representative structure is bovine GRK1, PDB ID, 3C4W. *P<0.05; **P<0.01; ***P<0.001, n.s. not significant (differences relative to WT GRK1).
