# Supplementary material for: Molecular assembly of rhodopsin with G protein-coupled receptor kinases
Source: Cell Res. 2017 May 19;27(6):728–47. doi: 10.1038/cr.2017.72 (PMC5518878; doi:10.1038/cr.2017.72)
Supplement: Supplementary information, Figure S6 — HDX analysis of human GRK1 in the absence and presence of rhodopsin. [file cr201772x6.pdf]

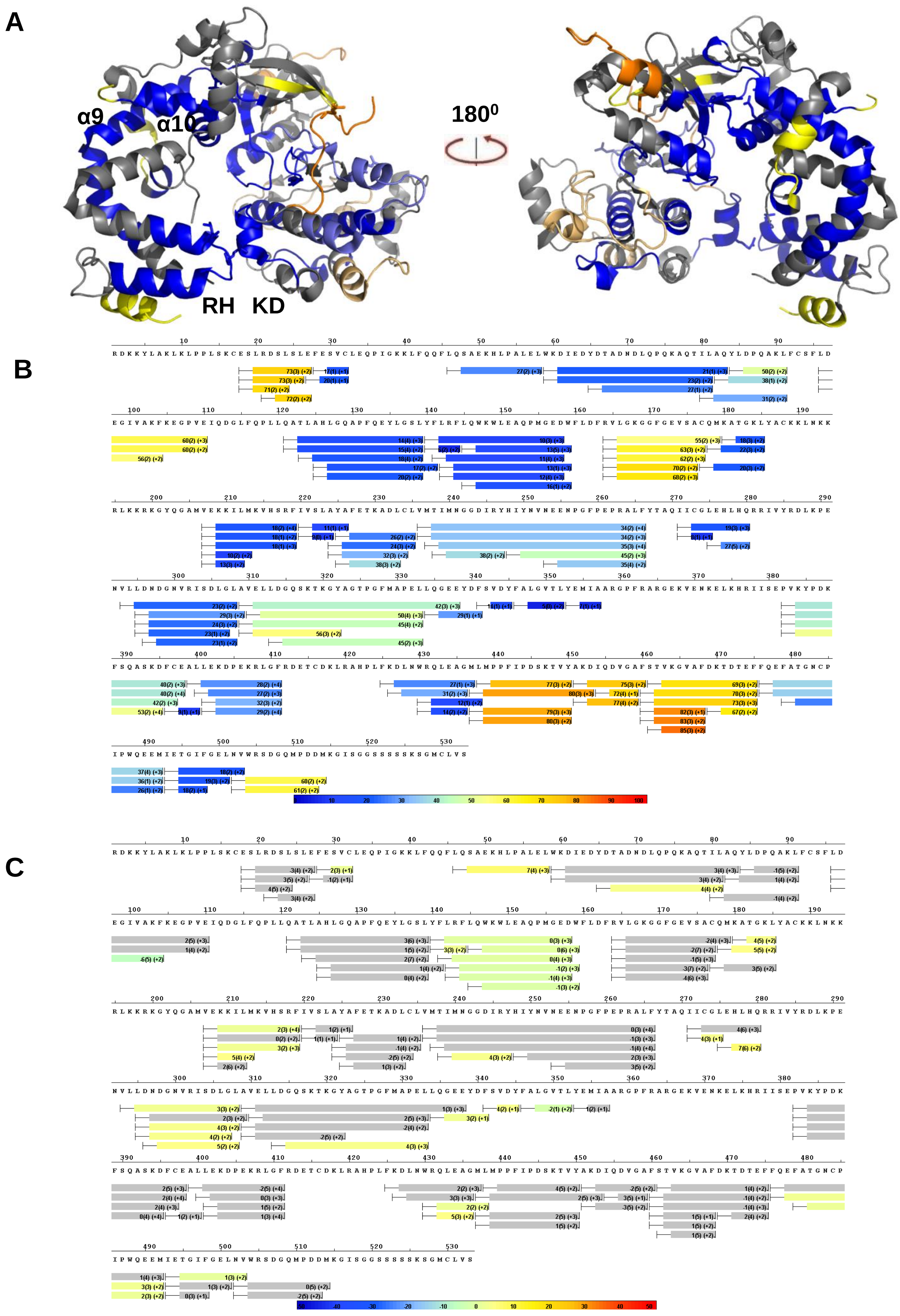
A
α9
α10
RH
KD
180⁰
B
C

Supplementary information, Figure S6. HDX analysis of human GRK1 in the absence and presence of rhodopsin. (**A**) Apo GRK1 HDX heat map overlaid onto a representative structure of bovine GRK1 (PDB ID 3C4W). (**B**) HDX map of apo GRK1. The bars below the GRK1 sequence represent the peptide fragments resolved by mass spectrometry and the colors of the bars indicate the average % of deuterium uptake over a 1hr time period (color code at bottom). (**C**) HDX perturbation map between rhodopsin-bound GRK1 and free GRK1. The bars below the GRK1 sequence represent the peptide fragments resolved by mass spectrometry and the colors of the bars indicate the relative % difference in deuterium exchange upon rhodopsin binding (color code at bottom).
