# Supplementary material for: Molecular assembly of rhodopsin with G protein-coupled receptor kinases
Source: Cell Res. 2017 May 19;27(6):728–47. doi: 10.1038/cr.2017.72 (PMC5518878; doi:10.1038/cr.2017.72)
Supplement: Supplementary information, Figure S7 — Mutations of residues of the α4-α6 bundle of the RH domain of GRK1 do not inhibit rhodopsin binding in Tango assay. [file cr201772x7.pdf]

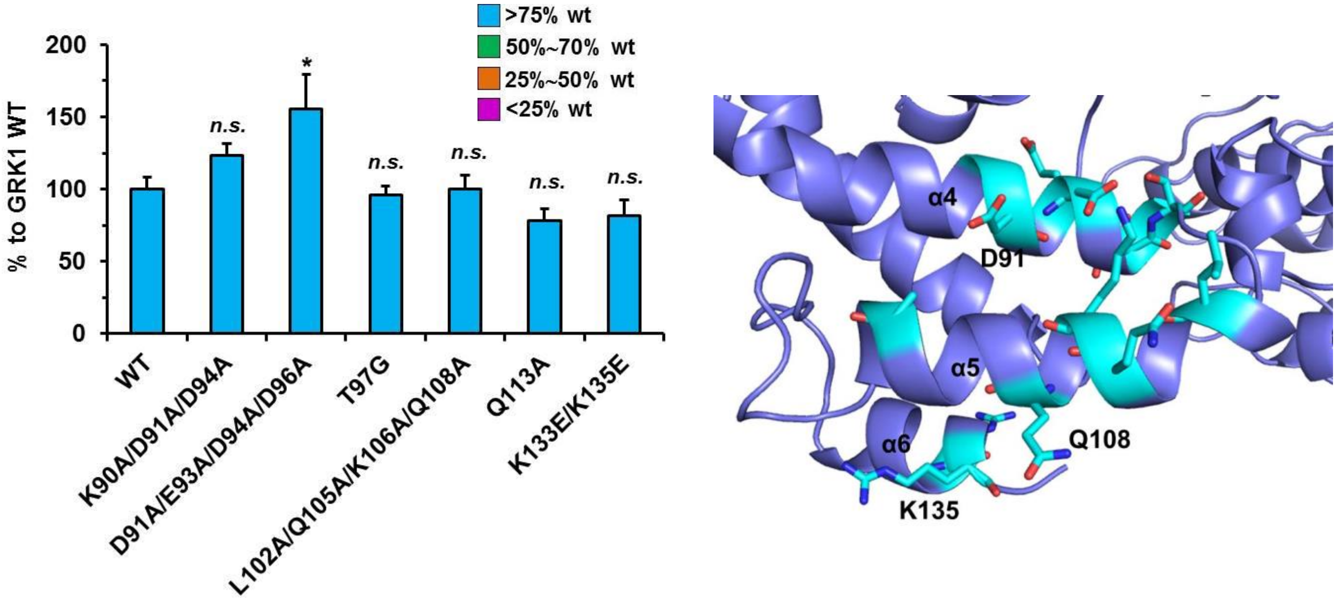

Supplementary information, Figure S7. Mutations of residues of the α4-α6 bundle of the RH domain of GRK1 do not inhibit rhodopsin binding in Tango assay. Data were plotted as percent of wild type GRK1 binding capacity (Y-axis).. Representative structure is bovine GRK1, PDB ID, 3C4W. n.s. not significant (differences relative to WT GRK1).
