# Supplementary material for: Molecular assembly of rhodopsin with G protein-coupled receptor kinases
Source: Cell Res. 2017 May 19;27(6):728–47. doi: 10.1038/cr.2017.72 (PMC5518878; doi:10.1038/cr.2017.72)
Supplement: Supplementary information, Figure S8 — Biochemical properties of BRIL-rhodopsin-GRK5 fusion protein. [file cr201772x8.pdf]

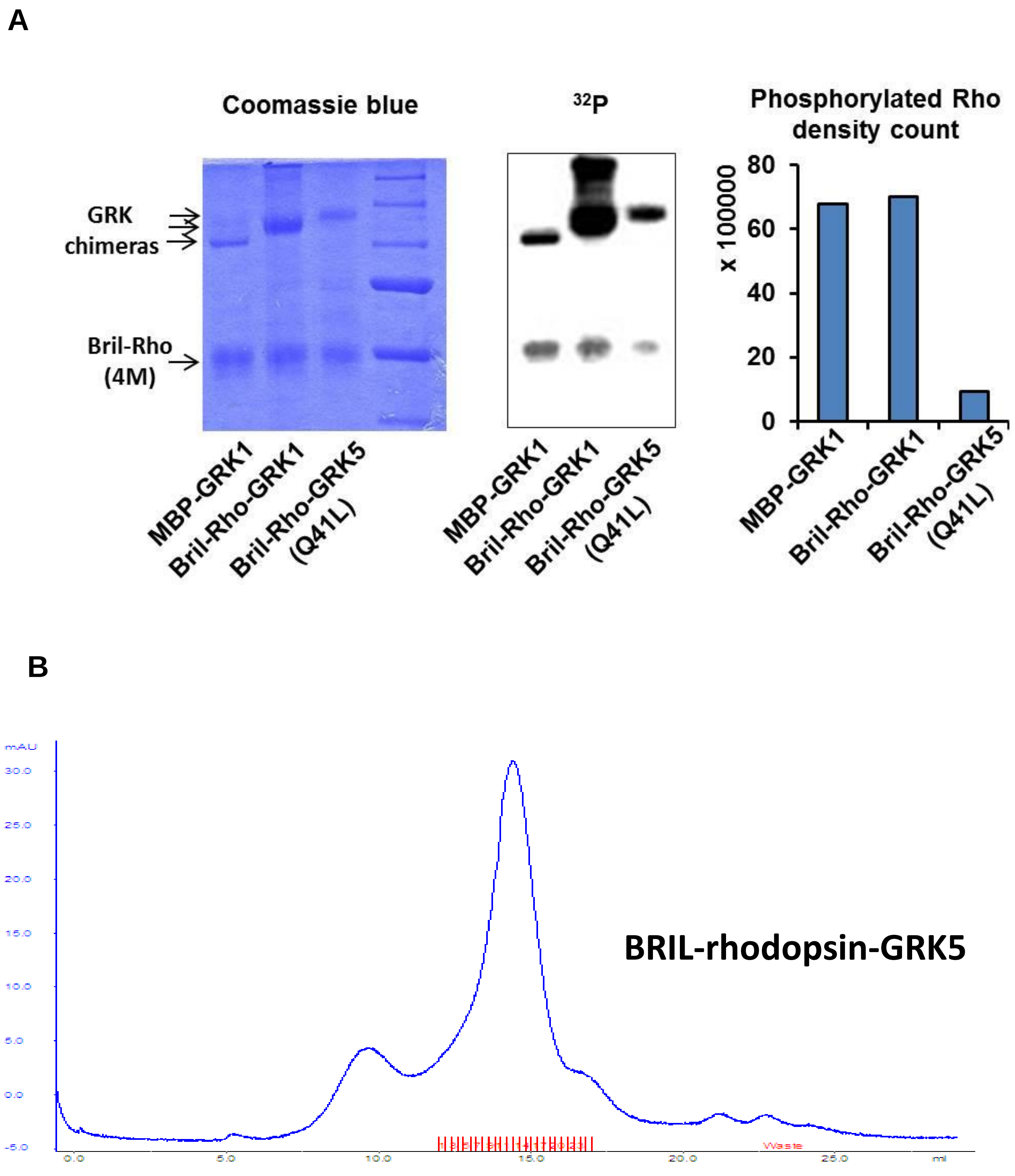

Supplementary information, Figure S8. Biochemical properties of BRIL-rhodopsin-GRK5 fusion protein. (**A**) Direct kinase assay of BRIL-rhodopsin-GRK fusion proteins. (**B**). Size column profile of BRIL-rhodopsin-GRK5 fusion protein.
