# Supplementary material for: Molecular assembly of rhodopsin with G protein-coupled receptor kinases
Source: Cell Res. 2017 May 19;27(6):728–47. doi: 10.1038/cr.2017.72 (PMC5518878; doi:10.1038/cr.2017.72)
Supplement: Supplementary information, Table S1 — A summary of rhodopsin binding capacities of all GRK1 mutant proteins. [file cr201772x9.pdf]

GRK1 mutations	% to WT	SD
WT	100	8.345
L6A	47.813	0.217
F15A	61.680	12.553
S27A	71.112	10.464
K33E	75.733	9.501
K33A/K34A	78.819	1.354
K34A	77.661	9.902
Y35A	43.078	8.806
K38A	79.803	4.966
L39G	4.937	0.220
K40C	95.791	22.952
L39G/L41G/P42A	4.208	1.190
L41G	5.042	1.685
P42A	23.965	6.063
P42A/L71A	3.077	0.754
K46A/E48A	104.034	3.853
R51A	105.294	3.718
D52A	101.134	17.714
D52RS55R	99.903	12.134
E59A	133.490	13.567
L63P/Q118A	3.042	0.282
L71A	5.024	0.496
Q74A	63.787	24.978
Q77A	135.201	18.895
E80R	95.895	11.276
K90A/D91A/D94A	123.507	8.319
D91A/E93A/D94A/D96A	155.729	23.879
T97G	96.039	6.232
L102A/Q105A/K106A/Q108A	99.997	9.885
Q113A	78.278	8.209
D116R	82.005	4.923
D116R/P117A/Q118A/K120E	22.692	4.825
P117A	67.804	3.416
P117A/Q118A	102.552	11.544
Q118A	47.113	13.292
K120E	69.792	8.131
F122E	112.938	21.223
S124L	102.228	5.515
F125E	67.980	7.725
K133E/K135E	81.839	10.368
Q159A	65.931	2.995
Q163A/E164A	99.304	7.937
L169A	38.113	2.803
Y170A/Q176A	4.827	0.604
Y170A/W177A	3.536	0.522
L172C	13.718	5.375
R173A	14.100	4.008
Q176A	5.492	1.434
W177A	4.315	0.359
W189A	77.089	11.128
D192A	76.431	13.875
R194A	103.562	26.019
K198A	78.040	15.444
T212G	20.413	6.389
K219A	6.523	0.554
K219R	10.489	1.672
K220A	30.229	3.398
K227A	66.898	5.452
E238A	24.688	10.110
K244A	69.190	17.323
Y255A	46.392	2.145
R276A	59.737	16.316
D317A	17.413	3.131
D335A	75.141	1.513
R461A	84.965	16.976
P470A/P471A/P474A	6.858	1.351
K477A	70.520	15.564
V479A	86.458	18.399
Y480D	43.437	4.574
F505A	50.989	6.333
F509A	21.494	0.315
E521A	35.620	6.316
F528E	6.395	1.964
G529A	125.049	8.467
L531E	29.493	1.931
R535E/S536E	56.167	8.848
C560A	95.380	5.433

>75%
50%-75%
25%-50%
<25%

Supplementary information, Table S1. A summary of rhodopsin binding capacities of all GRK1 mutant proteins. Data were plotted as percent binding capacity relative to wild type GRK1.
